# A novel modular modeling approach for understanding different electromechanics between left and right heart in rat

**DOI:** 10.3389/fphys.2022.965054

**Published:** 2022-09-13

**Authors:** Nari Kim, Julius D. Pronto, David P. Nickerson, Andrew J. Taberner, Peter J. Hunter

**Affiliations:** ^1^ NLRL for Innovative Cardiovascular Engineering, Department of Physiology, College of Medicine, Inje University, Busan, South Korea; ^2^ Cardiovascular and Metabolic Disease Center, Inje University, Busan, South Korea; ^3^ Auckland Bioengineering Institute, The University of Auckland, Auckland, New Zealand

**Keywords:** modular modeling, electromechanics, interventricular difference, stress, Ca^2+^ modulation

## Abstract

While ion channels and transporters involved in excitation-contraction coupling have been linked and constructed as comprehensive computational models, validation of whether each individual component of a model can be reused has not been previously attempted. Here we address this issue while using a novel modular modeling approach to investigate the underlying mechanism for the differences between left ventricle (LV) and right ventricle (RV). Our model was developed from modules constructed using the module assembly principles of the CellML model markup language. The components of three existing separate models of cardiac function were disassembled as to create smaller modules, validated individually, and then the component parts were combined into a new integrative model of a rat ventricular myocyte. The model was implemented in OpenCOR using the CellML standard in order to ensure reproducibility. Simulated action potential (AP), Ca^2+^ transient, and tension were in close agreement with our experimental measurements: LV AP showed a prolonged duration and a more prominent plateau compared with RV AP; Ca^2+^ transient showed prolonged duration and slow decay in LV compared to RV; the peak value and relaxation of tension were larger and slower, respectively, in LV compared to RV. Our novel approach of module-based mathematical modeling has established that the ionic mechanisms underlying the APs and Ca^2+^ handling play a role in the variation in force production between ventricles. This simulation process also provides a useful way to reuse and elaborate upon existing models in order to develop a new model.

## Introduction

Mathematical modeling is a powerful technique that can improve one’s understanding of normal and pathophysiology by incorporating a wealth of biological data that many researchers have accumulated. Using this comprehensive set of experimental data, many biophysical cell models have been developed to investigate the effect of subcellular changes on overall organ function, and many frameworks for simulation have been proposed. Most researchers want to share existing models and extend them into large-scale models that address the complexity at the whole cell level. As complex models need to be developed efficiently, many kinds of simulation tools have been developed. Among them, CellML, a model encoding format, has a couple of strengths. *1*) It uses MathML to represent the mathematics of the model in a way that both humans and computers can read (Terkildsen et al., 2008). *2*) The component instance and encapsulation layers (Cooling et al., 2016) allow modular configuration of the model to provide model scalability and model reuse.

The treatment of cardiovascular disease is shifting the paradigm from correcting a patient’s deranged hemodynamic factors to blocking the most potent active signaling pathways in the disease. Accordingly, the strategy of cardiovascular disease research has turned to exploring ways to improve outcomes by modulating active signaling pathways through identification of relevant biochemical mechanisms, and most drug targets are membrane-bound proteins for cell signaling. Cellular signaling, on the other hand, integrates the behavior of many biological components, so it is not possible to gain mechanistic insights solely from knowledge of individual system components. Validated computational models can be dissected in time, physical space or parametric space, allowing the cellular and subcellular processes underlying normal or abnormal tissue and organ function to be validated at levels that cannot be achieved experimentally (Clayton, 2001). Therefore, there is a need for a computer model to investigate the physiological and pathophysiological mechanisms of normal and abnormal cardiovascular function in parallel with experimental studies.

The differences in the contractile performance between the left ventricle (LV) and the right ventricle (RV) based on their geometries have been examined in normal heart as well as diseased heart. In addition, distinct differences in the adaptive response of each ventricular cell have been observed in pathological conditions. However, clinical approaches to improving cardiac performance can be equally applicable to the LV and the RV in various forms of heart disease and are based on a general understanding of the laws that define cardiac mechanics. In practice, appropriate therapies for LV dysfunction have been shown to be not necessarily ideal for RV dysfunction (Walker and Buttrick, 2009). Therefore, a comprehensive understanding of the differences in the contractile performance between the LV and the RV myocytes in the normal heart as well as the diseased heart is important for improvement of existing therapeutics for various cardiovascular diseases.

Myocardial wall stress is the integration of the tension of individual myocardial fibers and is determined by the left ventricular cavity and wall dimensions (Laplace law) as well as ventricular pressure (Chowienczyk and Shah, 2012). These ventricular pressures and volumes show the length dependence of the contractile force (Frank-Starling effect) and are interdependent as a result of the inverse relationship between force generation and shortening velocity in the muscle. Thus, different myocardial wall stresses (or wall tensions) may result in different contractile properties in the LV and RV of a normal heart. During cardiac contraction, the wall stress increases as the myofilaments undergo cross-bridge cycling. The sequence of this excitatory-contraction coupling is similar between LV and RV, but the relative contributions of ion channels vary, affecting their action potential and ultimately their contractile behavior (Kim et al., 2010). In addition, ventricular wall stress determines oxygen consumption, cardiac hypertrophy response, and hepatic fibrosis. Quantification of ventricular wall stress is necessary to understand normal and pathological ventricular mechanics (Yin, 1981). Clinically, normalization of wall stress in volume/pressure-loaded heart disease has been considered a feedback mechanism that governs the rate and extent of development of ventricular hypertrophy (Hood et al., 1968; Grossman et al., 1975; Alter et al., 2012). Therefore, understanding the ventricular wall stress can provide important insights into the underlying ventricular mechanics and energetics in compromised hearts.

In this study, we performed quasi-isometric contraction experiments to compare different stresses in LV and RV muscles and Ca^2+^ imaging experiments for comparing different Ca^2+^ modulation between LV and RV myocytes. Then we implemented a mathematical model, first building a component model of individual transmembrane currents involved in the excitation-contraction (EC) coupling process, testing whether they function as an independent working model, and then combining them into a new integrative model of rat ventricular myocytes. The integrative model as well as modular model constituents were validated based on the experimental data and aided in drawing conclusions.

## Materials and methods

### Animal and ethics statement

Male Wistar rats (280–380 g) were used in this study. All experimental procedures were performed according to the requirements of the Animal Ethics Committee of the University of Auckland (Approval R595 and R787) and the National Animal Ethics Advisory Committee (The Animal Welfare Act 1999; Schedules 1 to 7) as well as the protocol approved by the Institutional Review Board of Animals of Inje University College of Medicine.

### Experimental procedures

Each experimental procedure was carried out with the same ways of previous studies. Briefly, the rats were anesthetized with isoflurane mixed in 100% O_2_. The heart was quickly excised after cervical dislocation, placed in cold saline solution, and then perfused with an oxygenated solution of either low-calcium Tyrode’s solution containing 2,3-butanedione monoxime (BDM) or normal Tyrode’s solution, for trabecular muscles preparation or single cardiomyocytes preparation, respectively. In mechanical experiments, we measured active stress production of trabecular muscles from the LV and RV free wall using our mechanical testing device (Han et al., 2009). In Ca^2+^ transient experiments, we measured Ca^2+^ ion flux of single cardiomyocytes from the left and right midventricular wall using the ion-specific probe Fluo-4 AM (Invitrogen, Eugene, OR, United States) (Kim et al., 2012). In electrophysiological experiments, we measured action potentials of single cardiomyocytes from the left and right midventricular wall using the patch-clamping technique (Kim et al., 2010).

### Model development

EC coupling is the process that links an action potential (electric excitation of cell membrane) to contraction (shortening and force development) of the heart. Action potential (AP) is caused by different ions crossing the plasma membrane through various ion channels and transporters. This electric excitation of the sarcolemma/T-tubule induce Ca^2+^ release from sarcoplasmic reticulum (SR) *via* Ca^2+^-induced Ca^2+^ release (CICR). Then free Ca^2+^ binds to the troponin complex, which facilitates the interaction of actin and myosin and contraction takes place. A schematic diagram of major ion currents and Ca^2+^ handling proteins, myofibrillar proteins are shown in Supplementary Figure S1. All the ionic processes and compartments in the whole cell model were used as modules and addressed briefly as described below. Our model retains the fundamental features of cardiomyocyte electrophysiology originally described by Pandit *et al.* (Pandit et al., 2001), detailed Ca^2+^ dynamics originally described by Hinch *et al.* (Hinch et al., 2004), and active contraction originally described by Niederer *et al.* (Niederer et al., 2006) in the rat heart: *1*) the model formulations of the Na^+^ channel, the Ca^2+^-independent K^+^ channel, the steady-state K^+^ channel, the inward rectifier K^+^ channel, the hyperpolarizing-activated channel, the background K^+^ channel, the background Na^+^ channel, the Na^+^-K^+^ pump, and the calmodulin were extracted from the Pandit *et al.* model; *2*) the model formulations of the Ca^2+^ release unit, the Ca^2+^ pump, the Na^+^-Ca^2+^ exchanger, and the background Ca^2+^ channel were extracted from the Hinch *et al.* model; *3*) the model formulation of the troponin, the tropomyosin, and the cross-bridge kinetics were extracted from the Niederer *et al.* model; *4*) the model parameters and variables were adjusted to match the experimental observations, as described in the section below. The model also accounts for dynamic changes in ionic concentrations and fluxes during the action potential.

Based on our design principles for modularity in CellML (Cooling et al., 2016), we disassembled the components of the three separate models listed above and to produce individual working and testable modules. These modules which encompass variables and mathematics were implemented as CellML components and were independently tested and validated. Individual components are available online (https://models.physiomeproject.org/workspace/25c): INa_Pandit, Ito_Pandit, Iss_Pandit, IK1_Pandit, If_Pandit, IBNa_Pandit, IBK_Pandit, INaK_Pandit, CaRU_Hinch, ICaPump_Hinch, INCX_Hinch, IBCa_Hinch, ISRCaLeak_Hinch, ISERCA_Hinch, Icalmodulin_New, Itroponin_NSH, Itropomyosin _NSH, CrossBridge_NSH. In turn we combined the validated modules into a new integrative model of a rat ventricular myocyte using standard unit and universal constant modules. This model was validated against the experimental data. CellML text code for units are illustrated in Supplement 1. All model equations and parameter values are provided in Supplement 2. All simulations were performed using the OpenCOR desktop application version 0.3 (Garny and Hunter, 2015).

### Formulation and validation of the individual model modules

The electrophysiological behavior can be described by the ordinary differential equation:

$$\frac{dV}{dt}=-\frac{I_{ion}+I_{stim}}{C_{m}}$$

where *V* is voltage, *t* is time, $I_{ion}$ is the sum of all transmembrane ionic currents, $I_{stim}$ is the externally applied stimulus current, and $C_{m}$ is cell capacitance per unit surface area.

#### Na^+^ current (*I*_Na_) module

**FIGURE 1:**
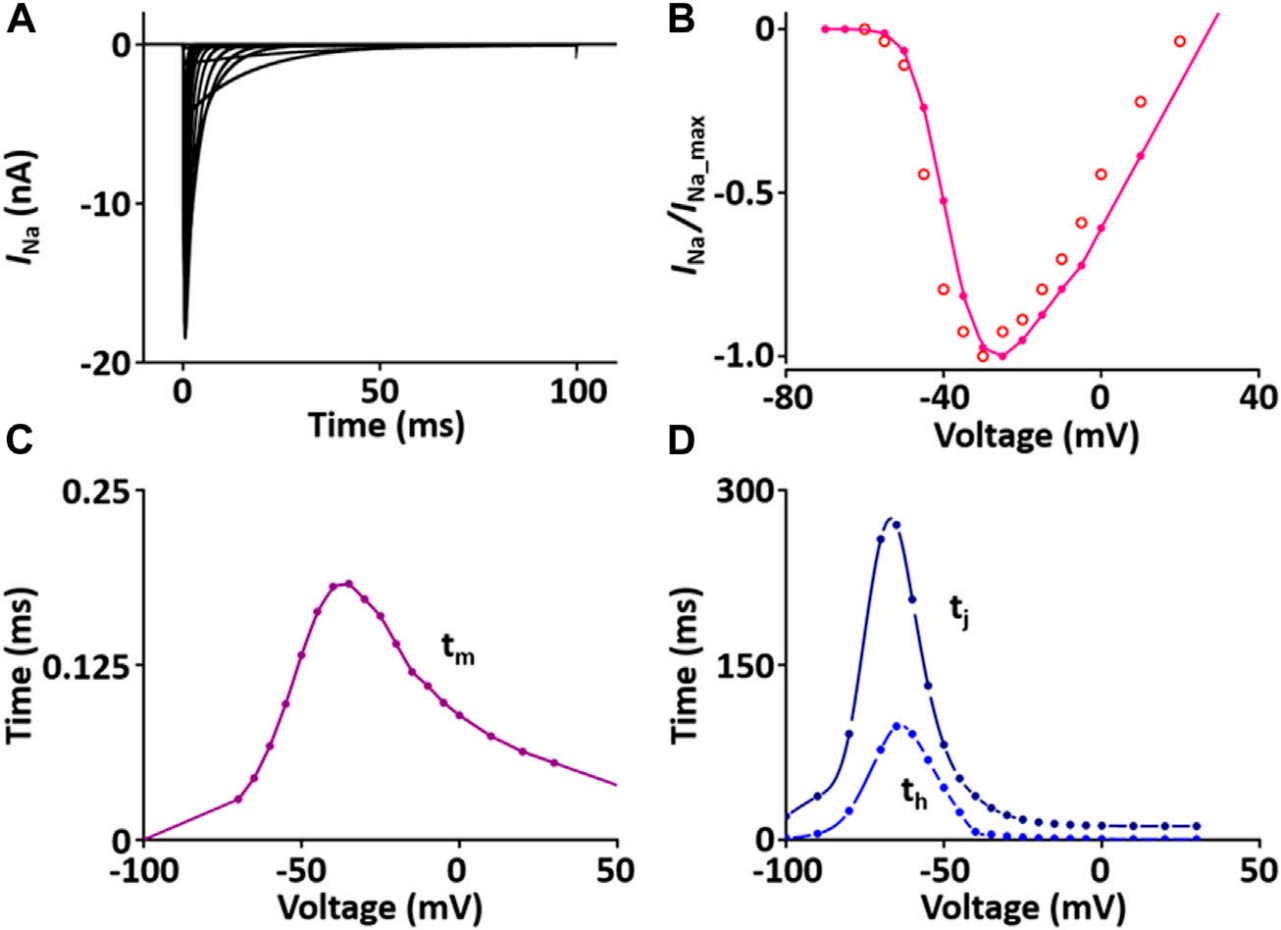
Verification of *I*_Na_ implementation (Kim, 2018). **(A)** Simulated current traces of the Na^+^ channel. **(B)** Normalized simulated *I-V* relationship of *I*_Na_ (o represents experimental data from Lee et al., 1999). **(C)** Simulated activation time constant (τ_m_). **(D)** Simulated inactivation time constants (τ_h_, τ_j_).

*I*_Na_ is responsible for upstroke of the AP. The formulation of *I*_Na_ model is from Pandit et al. (2001) and the CellML text code for *I*_Na_ model is as shown in Supplement 1. *I*_Na_ kinetics were reported to be similar across different species, giving us a rationale to use these variables: the steady-state activation and inactivation curves, the normalized peak current-voltage (*I-V*) relationship, and the maximum Na^+^ conductance (*g*_Na_) are based and adjusted from the rat study (Lee et al., 1999); the time constants for activation (*τ*_m_) and inactivation (*τ*_h_, *τ*_j_) used as model parameters were adapted from the guinea pig study (Luo and Rudy, 1994) and scaled. The m-gate, h-gate, and j-gate of three gates for *I*_Na_ were encapsulated and embedded inside the sodium channel. We used OpenCOR, with end point 40 ms and Point interval 0.1 ms, to solve the equations for *I*_Na_ under several voltage steps in one fixed condition with [Na^+^]_o_ = 145 mM and [Na^+^]_i_ = 11.28 mM. We obtained numerical values for all variables and saved them as comma-separated values (CSV) files and then plotted whole-cell current traces, the *I*-*V* relationship and time constants, which corresponded well with published results (Figure 1).

Each module was modeled in the same way and the values were compared with the experimental data or the results of the other studies.

#### Ca^2+^-independent transient outward K^+^ current (*I*_to_) module

**FIGURE 2:**
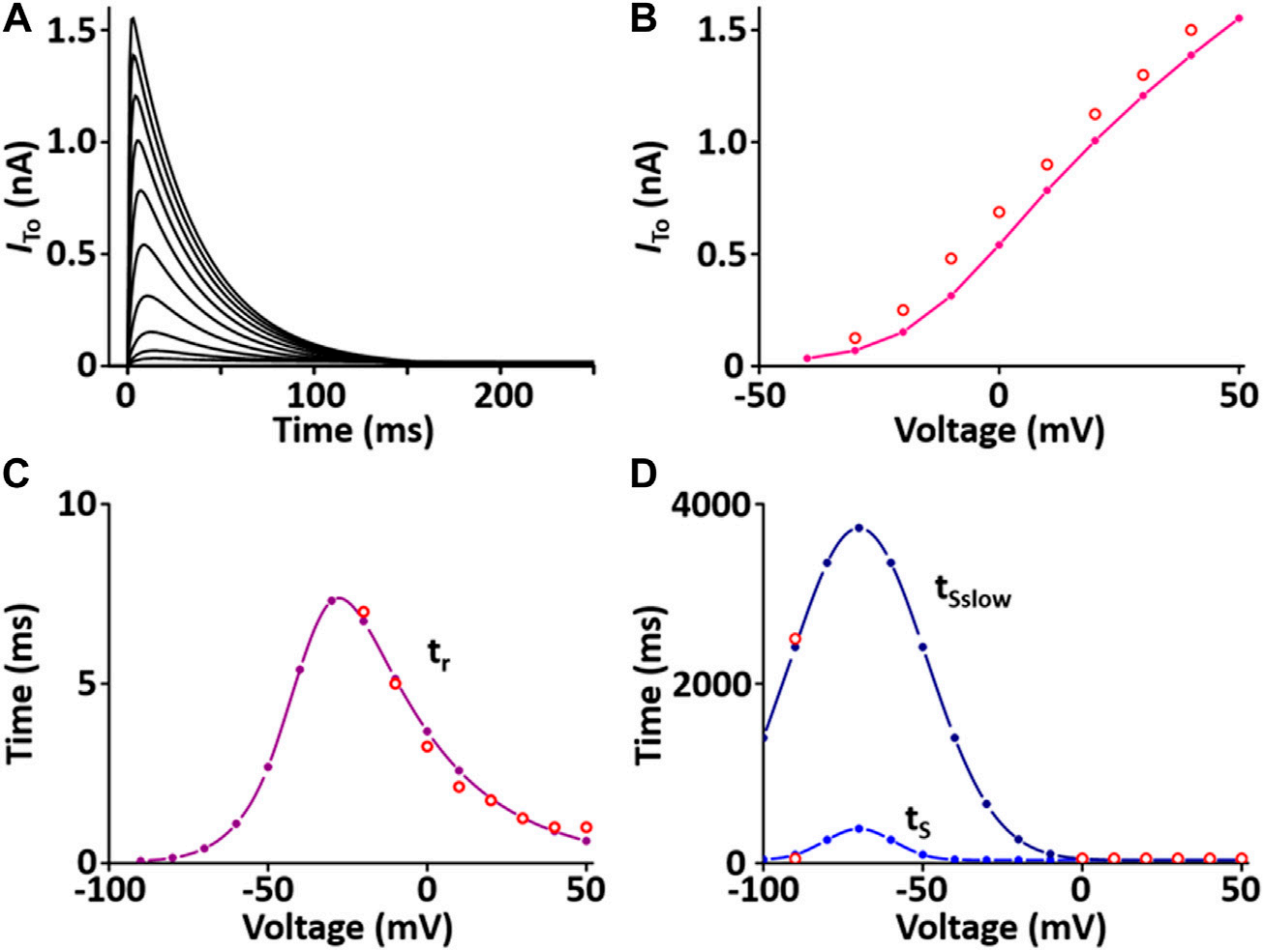
Verification of *I*_to_ implementation (Kim, 2018). **(A)** Simulated current traces of Ca^2+^-independent transient outward K^+^ channel. **(B)** Simulated *I-V* relationship for *I*_to_ (pink line). This simulated result is in close agreement with representative experimental result (o, Clark et al., 1995). **(C)** Simulated activation time constant (τ_r_, purple line). This simulated result is in close agreement with the representative experimental result (o, Agus et al., 1991). **(D)** Simulated inactivation time constants (τ_S,_ τ_Sslow_, blue lines). This simulated result is in close agreement with the representative experimental result (o, Wettwer et al., 1993).

*I*_to_ contributes to the notch configuration of the AP. The formulation of the *I*_to_ model is from Pandit et al. (2001) and is as shown in Supplement 2. All values of the *I*_to_ model are based on individual experimental data from the rat heart: the steady-state activation and inactivation values are from Stengl et al. (Stengl et al., 1998); the time constant for activation from Angus et al. (Agus et al., 1991); the inactivation time constants from Wettwer et al. (Wettwer et al., 1993); and the recovery time constants from Volk et al. (Volk et al., 1999). In one fixed condition with [K^+^]_o_ = 5.4 mM and [K^+^]_i_ = 138.72 mM, the current traces and parameters of *I*_to_ corresponded well to published results (Figure 2).

#### Steady-state outward K^+^ current (*I*_ss_) module

**FIGURE 3:**
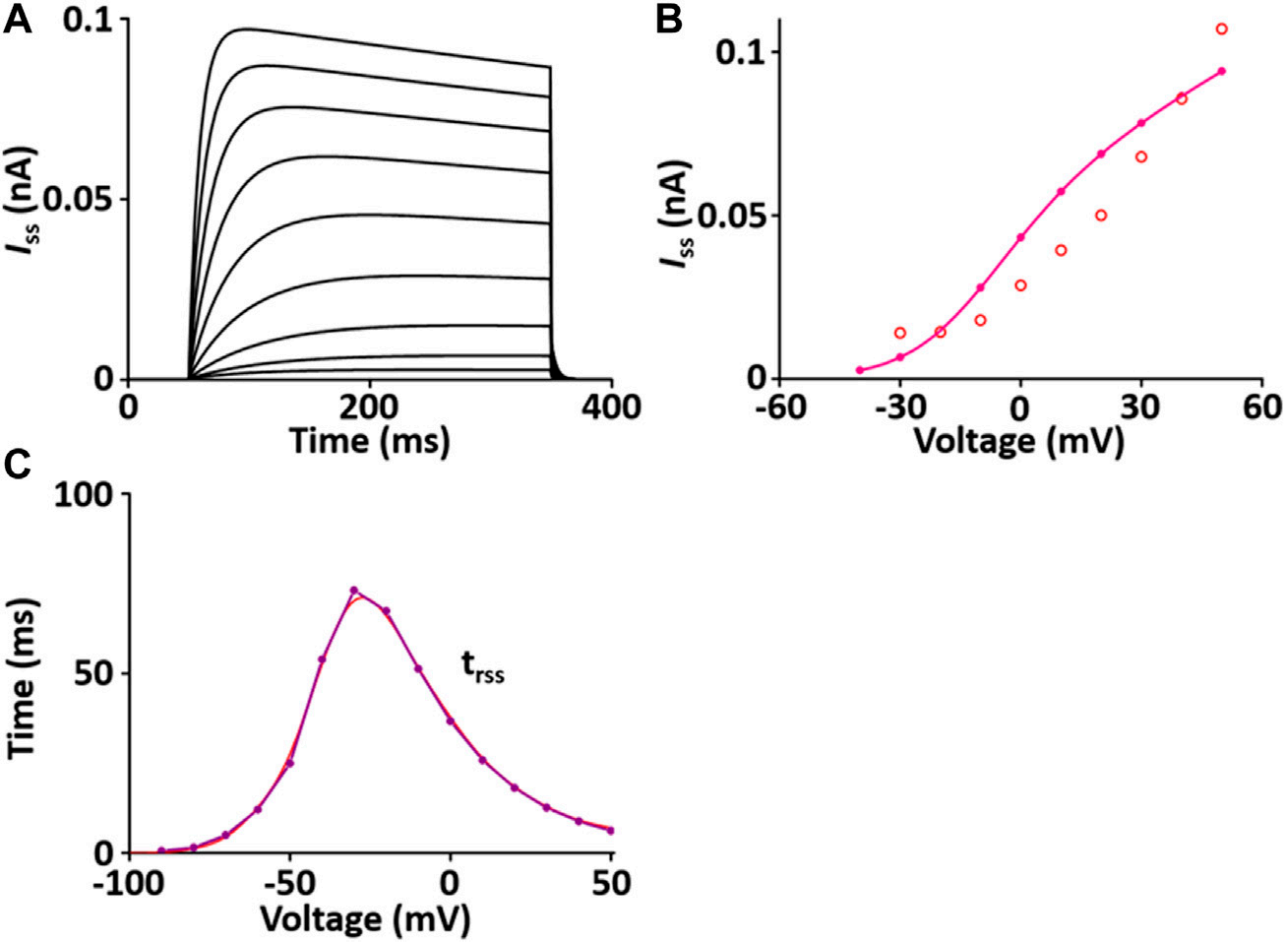
Verification of *I*_ss_ implementation (Kim, 2018). **(A)** Simulated current traces of the steady-state outward K^+^ channel. **(B)** Simulated *I-V* relationship of *I*_ss_ (pink line). This simulated result is in close agreement with the representative experimental result (o, Clark et al. (1995)). **(C)** Simulated activation time constant (τ_rss_, purple line). This simulated result is in close agreement with the representative experimental result (red line, Apkon and Nerbonne (1991)), which is 10 times slower than the corresponding one for *I*_to_ (Figure 3C).

*I*_ss_ contributes to the repolarization of the AP. The formulation of the *I*_ss_ model is from Pandit et al. (2001) and is as shown in Supplement 2. All values of the *I*_ss_ model are based on individual experimental data in the rat heart: the steady-state activation and inactivation values from Weis et al. (Weis et al., 1993); the time constant for activation, which is 10 times slower than that of *I*_to_, from Apkon and Nerbonne (Apkon and Nerbonne, 1991); the time constant for inactivation from Berger et al. (Berger et al., 1998). In one fixed condition with [K^+^]_o_ = 5 mM and [K^+^]_i_ = 140.45 mM, the current traces and parameters of *I*_ss_ corresponded well with published results (Figure 3). Values for *I*_ss_ were obtained at the end of a long (300 ms) depolarized voltage clamp pulse.

#### Inward rectifier K^+^ current (*I*_K1_) module

**FIGURE 4:**
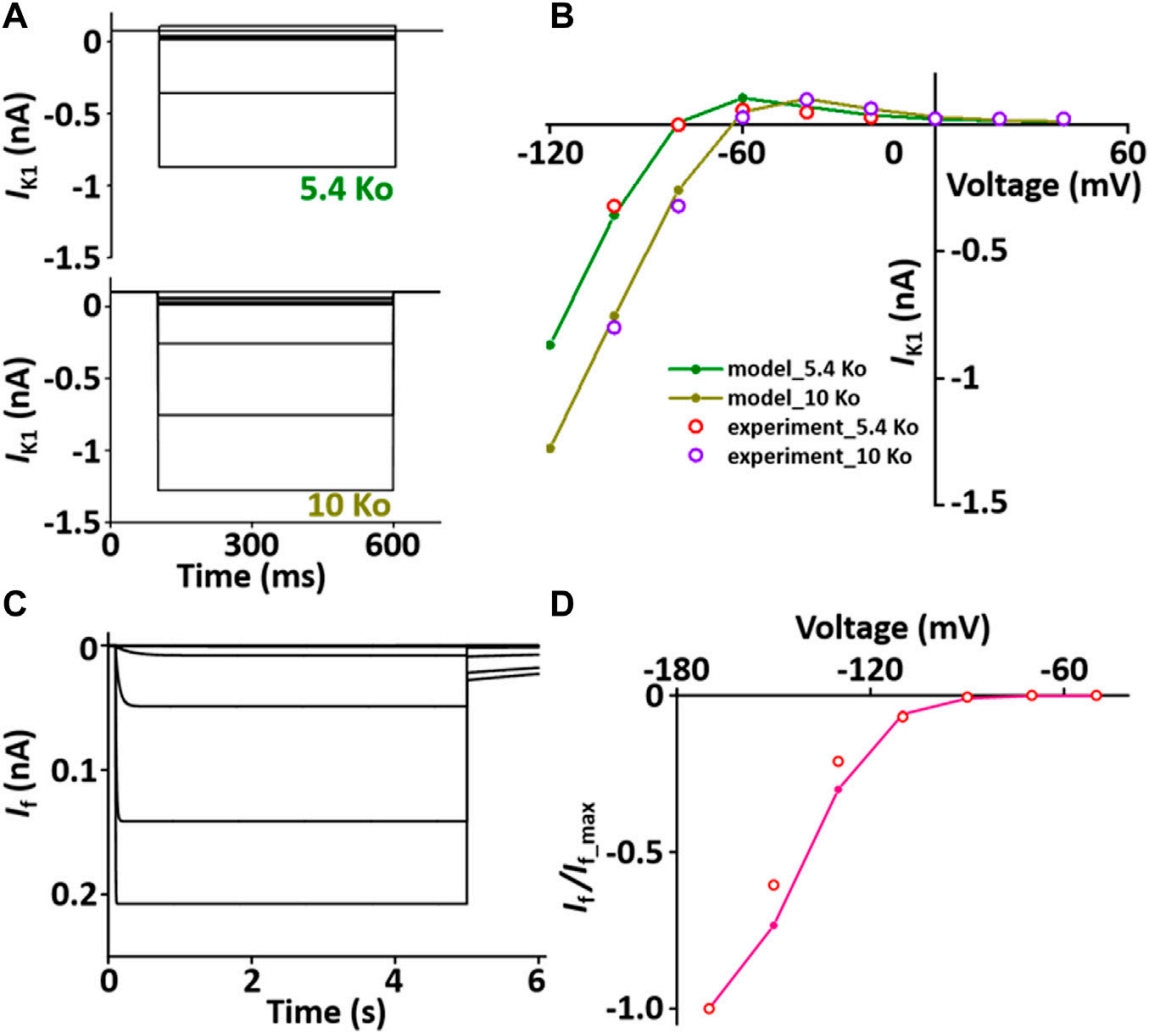
Verification of *I*_K1_ and *I*_f_ implementation (Kim, 2018). **(A)** Simulated current traces of the hyperpolarization-activated channel at extracellular K^+^ ion concentrations of 5.4 mM (5.4 Ko) and 10 mM (10 Ko). **(B)** Normalized simulated *I-V* relationship for *I*_K1_ (olive and dark yellow lines). This simulated result is in close agreement with the representative experimental results (experiment_5.4 Ko, experiment_10 Ko) which were digitized from Pandit et al. (2001)). **(C)** Simulated current traces of the hyperpolarization-activated channel. **(D)** Normalized simulated *I-V* relationship for *I*_f_ (pink line). This simulated result is in close agreement with the representative experimental result (o, Fares et al. (Fares et al., 1998)).

*I*_K1_ stabilizes the resting membrane potential. The formulation of *I*_K1_ model is from Pandit et al. (2001) and is as shown in Supplement 2. To characterize *I*_K1_ depending on [K^+^]_o_, current traces and *I*-*V* relationship of *I*_K1_ were verified in condition with [K^+^]_o_ = 5.4 mM or 10 mM, which corresponded well with published results (Figures 4A,B).

#### Hyperpolarization-activated current (*I*_f_) module

*I*_f_ is activated when the membrane is hyperpolarized from the resting potential and modulates the AP. The formulation of *I*_f_ model is from Demir et al. (Demir et al., 1994) and the values are based on experimental data from previous studies (Demir et al., 1994; Cerbai et al., 1996; Fares et al., 1998) and is as shown in Supplement 2. To verify the *I*_f_ model, the extracellular condition is to set [K^+^]_o_ and [Na^+^]_o_ at 25 and 30 mM, respectively, and the intracellular condition sets [K^+^]_i_ and [Na^+^]_i_ at 130 and 2 mM, respectively. Current traces and *I*-*V* relationship of *I*_f_ were corresponded closely with published results (Figures 4C,D).

#### Background Na^+^ current (*I*_BNa_) module

The formulation of *I*_BNa_ model is from the Pandit et al. (2001) model and is as shown in Supplement 2. The magnitude is adjusted based on experimental data from Demir et al. (1994).

#### Background K^+^ current (*I*_BK_) module

The formulation of *I*_BK_ model is from the Pandit et al. (2001) model and is as shown in Supplement 2. The magnitude is adjusted based on experimental data from Demir et al. (1994).

#### Na^+^/K^+^ pump current (*I*_NaK_) module

**FIGURE 5:**
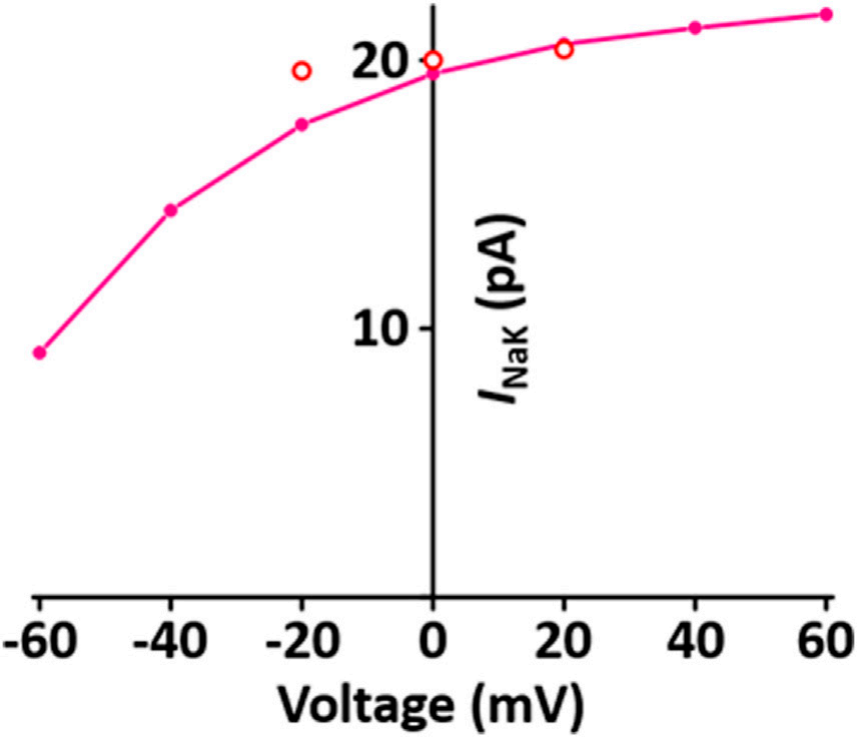
Verification of *I*_NaK_ implementation (Kim, 2018). Simulated *I-V* relationship for the sodium-potassium pump. o represents experimental data from Stimers and Dobretsov, 1998.

*I*_NaK_ directly produces a small potential difference, which causes the membrane potential to be negative. The formulation of *I*_NaK_ model is that of the Pandit et al. (2001) model adapted from earlier work by Luo and Rudy (1994) and is as shown in Supplement 2. When normalized to 100 pF, the *I*_NaK_ model was 0.195 A·F^−1^ at 0 mV, which is close to the value of 0.2 A·F^−1^ at 0 mV in the experimental data of Stimers and Dobretsov (Stimers and Dobretsov, 1998) (Figure 5).

#### Ca^2+^ release unit (CaRU) module

**FIGURE 6:**
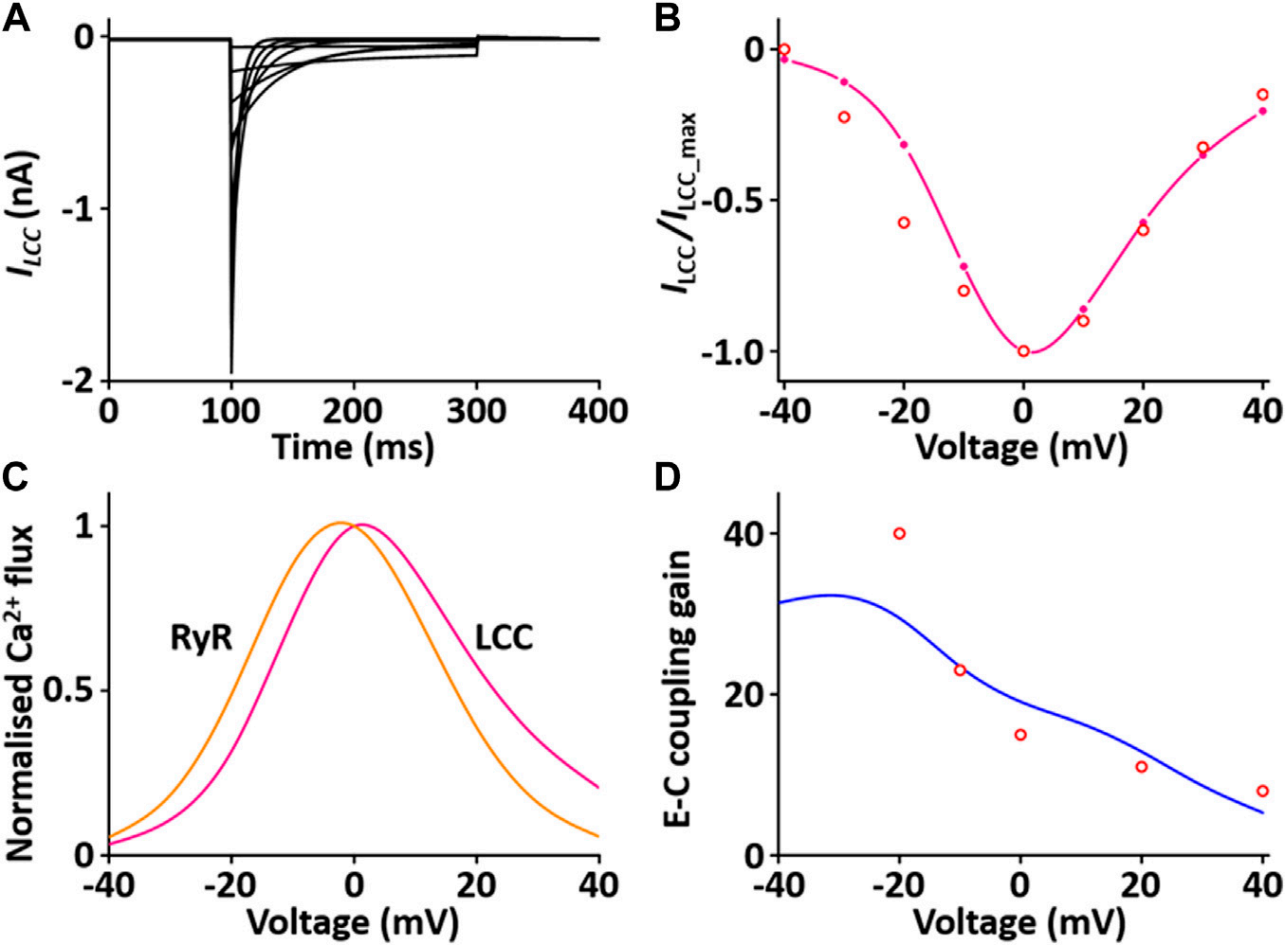
Verification of *I*_LCC_ and *I*_RYR_ implementation (Kim, 2018). **(A)** Simulated current traces for the LCC by a voltage step from −40 to 40 mV from a holding potential of −50 mV. **(B)** Normalized simulated *I-V* relationship for *I*_LCC_ (pink line). This simulated result is in close agreement with representative experimental result (o, Zahradnikova et al. (Zahradnikova et al., 2004)). **(C)** Simulated peak fluxes of LCC (pink line) and RYR (yellow line) as a function of membrane voltage. **(D)** Simulated EC coupling gain (blue line, maximum of RYR flux/maximum LCC flux) is in close agreement with representative experimental result (o, Wier at al. (Wier et al., 1994)).

The CaRU consists of one *L*-type Ca^2+^ channel (LCC) and ryanodine receptors (RYRs) and transports Ca^2+^ ions in the dyadic subspace between the *T*-tubules and the sarcoplasmic reticulum (SR). LCCs are located in sarcolemmal membrane and T-tubules and carries Ca^2+^ ions in the inward direction when the membrane is depolarized. RYRs are located in SR and release Ca^2+^ from SR *via* physical coupling to LCCs. These two channels induce to increase the cytosol Ca^2+^. The formulation of the LCC and RYRs models are from the Hinch et al. (2004) model, which is simplified by six coupled ordinary differential equations for Ca^2+^-induced Ca^2+^ release: *1*) the 3-state model of the LCC is based on Jafri et al. (Jafri et al., 1998) study and the 3-state model for RYR is based on Stengl et al. (1998) study; *2*) the 3-state LCC and the 3-state RYR models were combined to form a 9-state model, which was simplified further to produce a 4-state model of the CaRU. All parameters and functions of the CaRU model are shown in Supplement 2. To verify the CaRU model, the current traces and peak *I*-*V* relationship of the Ca^2+^ channel were obtained by various voltage steps from a holding potential of −50 mV in one fixed condition with [Ca^2+^]_o_ = 1.2 mM, which was compared with that measured experimentally. Simulated and measured currents were normalized to make the peak value of both equal to 1 at 0 mV, and both *I*-*V* curves are in close agreement. One feature of Ca^2+^ release is that the peak of *I*_RYR_ is shifted by ∼10 mV in the hyperpolarizing direction relative to that of *I*_LCC_, which is well simulated. Furthermore, EC coupling gain decreases as membrane voltage increases. This feature of simulated result corresponded well to published results (Figure 6).

#### Ca^2+^ pump current module

**FIGURE 7:**
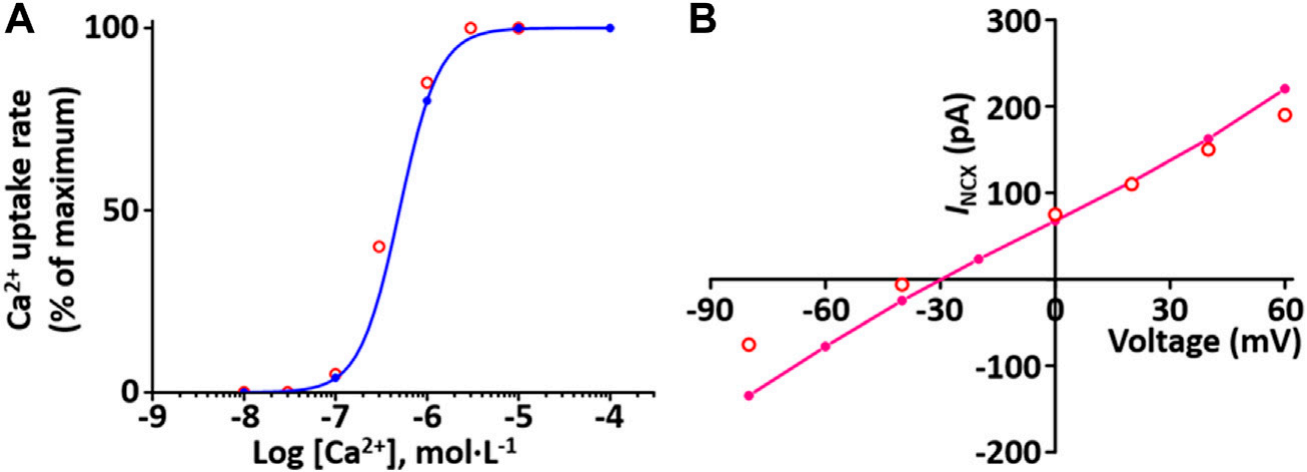
Verification of *I*_SERCA_ and *I*_NCX_ implementation (Kim, 2018). **(A)** The Ca^2+^ dependence of the relative rate of simulated SERCA activity is well plotted (blue line). This result is close agreement with representative experimental values (o, Lytton et al. (Lytton et al., 1992)). **(B)** Simulated *I-V* relationship for the NCX (pink line). This result is close agreement with representative experimental values (o, Li et al., 2013).

Sarcolemmal Ca^2+^ pump actively transports Ca^2+^ outward and SR Ca^2+^ pump actively transports Ca^2+^ from the cytosol into the SR lumen. These two pumps induce to decrease the cytosol Ca^2+^. The formulations of the SR Ca^2+^ pump, SERCA current (*I*_SERCA_) model and the sarcolemmal Ca^2+^ pump current (*I*_pCa_) model are from the Hinch et al. (2004) model and is as shown in Supplement 2. To verify the *I*_SERCA_ model, the Ca^2+^ dependence of SERCA activity was generated and compared with experimental data (Figure 7A)

#### Na^+^-Ca^2+^ exchanger current (*I*_NCX_) module

Na^+^-Ca^2+^ exchanger (NCX) exchanges 1 internal Ca^2+^ ion for three external Na^+^ ions *via* a membrane carrier. The formulation of the *I*_NCX_ model is from the Hinch et al. (2004) model and is as shown in Supplement 2. To verify *I*_NCX_, the extracellular condition set [Ca^2+^]_o_ and [Na^+^]_o_ at 1 and 140 mM, respectively, and the intracellular condition set [Na^+^]_i_ and free [Ca^2+^]_i_ at 20 and 1.2 mM, respectively, based on experimental conditions by Li et al. (Li et al., 2013). The simulated *I*_NCX_ model was generated, and the model output (Figure 7B) shows close agreement with the experimental results of Li et al. (2013).

#### Background Ca^2+^ current (*I*_BCa_) module

The formulations of the background Ca^2+^ current (*I*_BCa_) model is from the Hinch et al. (2004) model and is as shown in Supplement 2.

#### Calmodulin and troponin module

Ca^2+^ is buffered by calmodulin and troponin in the cytosol. The calculation of buffering by calmodulin is from the Pandit et al. (2001) model and by troponin current (*I*_TRPN_) model from the Niederer et al. (2006) model and is as shown in Supplement 2.

#### Tropomyosin module

Tropomyosin is situated in the actin groove and when it is shifted out of the actin groove, actin binds to myosin. The tropomyosin was characterized by the fraction of actin sites available for cross-bridge binding (*z*) and the formulation of *z* is from the Niederer et al. (2006) model and is as shown in Supplement 2.

#### Cross-bridge module

As myosin head attaches and detaches from myosin-binding site on the actin, it makes the actin filaments closer together, thus shortening the length of the sarcomere. These length changes of the myofibrils produce tension (cardiac contraction). The formulations of the isometric tension (T) is from the Niederer et al., 2006 model assuming a “fading memory,” and is as shown in Supplement 2. The fading memory model assumes that the current tension is influenced more by recent length changes than earlier length changes (Hunter et al., 1998) and phenomenologically explains the relationship between tension and cross-bridge kinetics. Here tension development can be separated into nonlinear static and linear time-dependent components and tension development associated with cross-bridge kinetics is proportional to *z*. The ratio of the isometric tension to the maximum tension at full activation for the same sarcomere length is equal to the ratio of *z* to the fraction of actin sites available at full activation for a given sarcomere length.

### Incorporating all components to the biophysical whole cell model

**FIGURE 8:**
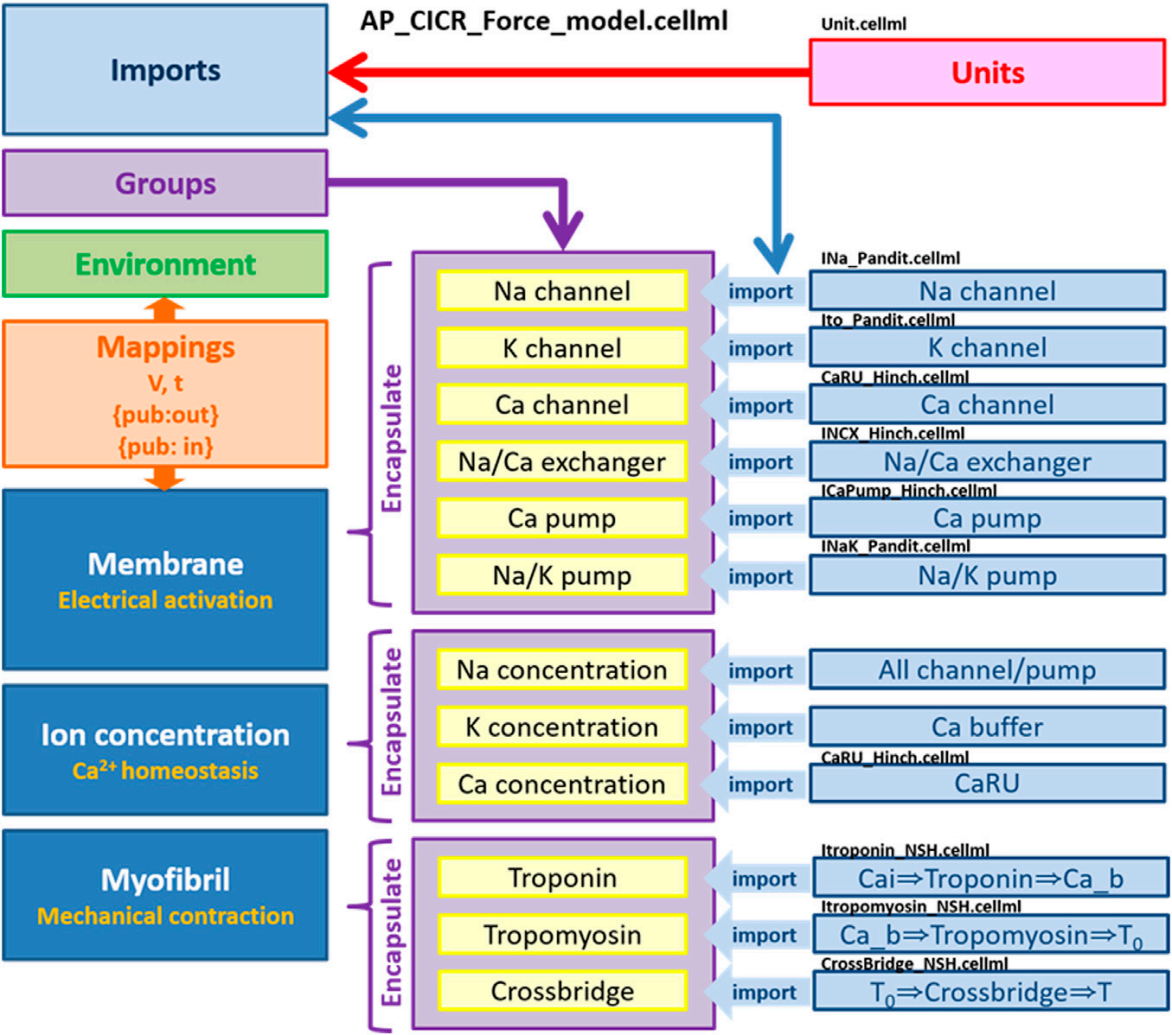
Overall structure of biophysical whole cell model (AP_CICR_Force_model) showing the encapsulation hierarchy (purple), the CellML model imports (blue) and the other key parts (units, components and mappings) of the top level CellML model (Kim, 2018).

We implemented all individual modules as CellML components and then, using the CellML model import facility in OpenCOR (http://www.opencor.ws/), we were able to compose the whole cell model, encompassing module variables and mathematics. Using the CellML model import facility allows to distribute these components among multiple files and to make in-memory copies of components from other files (Miller et al., 2010). The structure of the model, including separate files for units and component sets is shown in Figure 8.

When incorporating all components, we adjusted the *I*_LCC_ parameters based on previous results (Kim et al., 2010). The voltage at half-maximal activation was applied −15 mV and −10 mV for the LV and RV models, respectively. The formulation of *I*_*SERCA*_ obtained from the Hinch et al. (2004) model was adapted from earlier work by Jafri et al. (1998), so the maximum pump rate in their work is somewhat different from that in the rat. It is well known that the activity of SERCA is higher in the rat ventricle than in rabbit, ferret, dog, cat, guinea-pig and human ventricles (Bers, 2000). Therefore, $g_{SERCA}$ was increased by 20% to achieve similar activity for rat SERCA. The parameters of the troponin and the tropomyosin modules were adjusted to values appropriate for 37°C: temperature has some effect on the affinity of Ca^2+^ for TnC (McCubbin et al., 1980) and the affinity of Ca^2+^ for TnC was increased by 0.13 when temperature was increased from 21 to 37°C (Gillis et al., 2000); the wide range of Q_10_ values have been reported in order to reveal temperature dependence of force development in various muscles (Peiper et al., 1975; Stein et al., 1982; Janssen et al., 2002), we used three of Q_10_ value to parameterization of relaxation rates of tropomyosin.

## Results

### Verification of biophysical whole cell simulation: Action potential and Ca^2+^ dynamics

**FIGURE 9:**
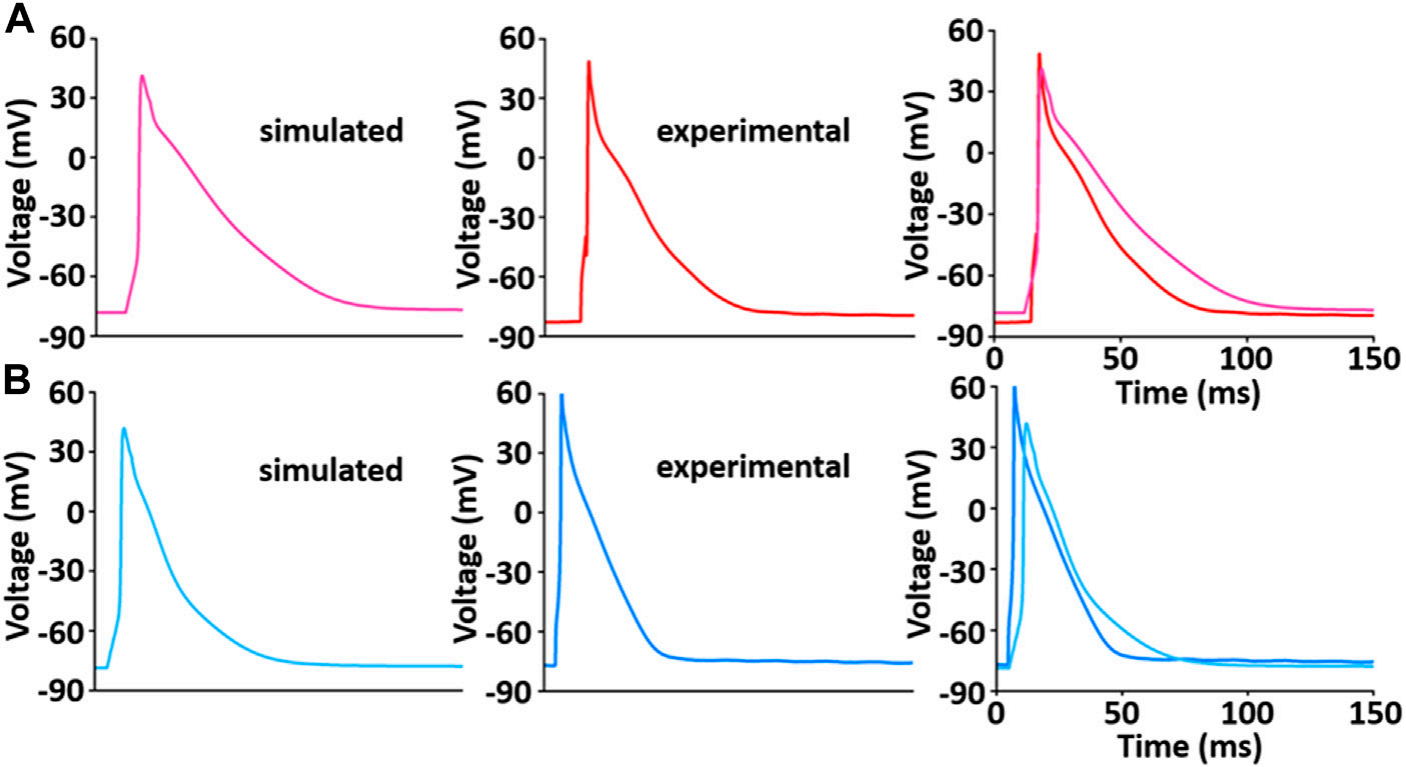
Model-generated LV **(A)** and RV **(B)** APs at 1 Hz and 22°C (Kim, 2018). Each simulated APs was validated with experimentally recorded LV **(A)** and RV **(B)** APs, respectively.

The whole cell model which was implemented *via* the steps described above shows the phenotype of typical AP waveforms, *I*_LCC_, and calcium transients in LV and RV myocytes (Supplementary Figure S2). Simulated AP waveforms are close agreement with our experimental recordings (Figure 9): the peak overshoot and duration of the simulated AP is very similar to the experimental recordings; the duration of simulated LV AP prolongs and the plateau phase of it is more prominent compared to the simulated RV AP. This result is in consistent with previous experimental results (Figure 9 and *see* Kaprielian et al., 2002; Kim et al., 2010; Stankovicova et al., 2000).

**FIGURE 10:**
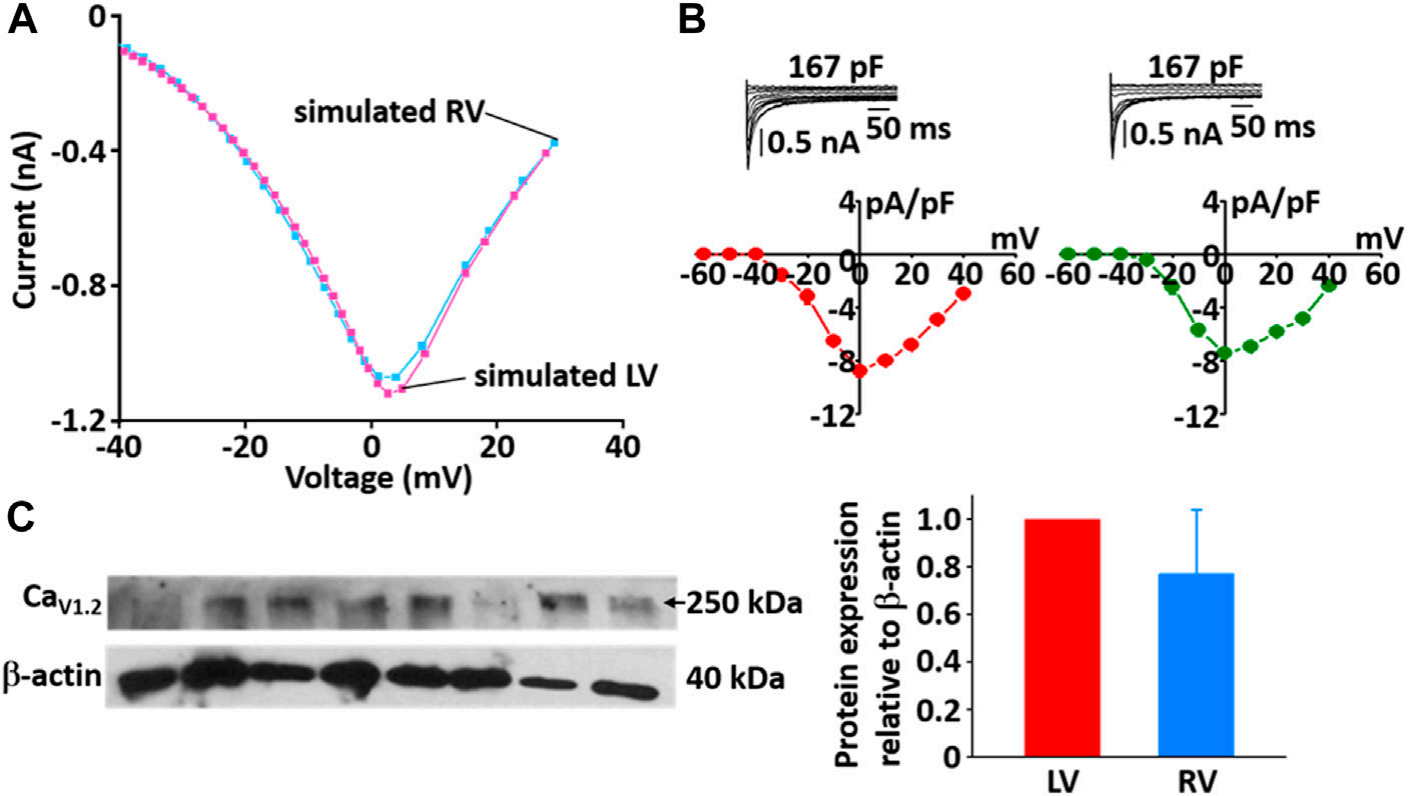
Comparison of *I*-*V* relationship of LCC in model **(A)** and experimental recordings **(B)** for LV and RV myocytes at 22°C (Kim, 2018). In panel **(B)**, inserts are representative ILCC traces from LV and RV myocytes and summary current-voltage relationships in LV (*n* = 5, red circles) and RV (*n* = 4, blue circles) myocytes, which from our previous study (Kim, Cannell et al., 2010). **(C)** Verification of protein expression of LCC proteins between LV and RV by immunoblotting. Representative blots and densitometric analysis of Ca_v1.2_ (250 kDa, *n* = 4) with β-actin (42 kDa) as housekeeping protein.

During the implementation of the cell model, one of the tuning parameters was the voltage at half-maximal activation of LCC, so it was necessary to compare the LCC characteristics between the LV and RV cells. The model-generated current-voltage (*I*-*V*) curves of LCC in LV and RV myocytes conformed well to previous experimental observation (Figures 10A,B). Peak current in LV myocytes was slightly larger than that in RV myocytes and peak *I*_LCC_ occurred at ∼0 mV in both LV and RV myocytes. However, as shown in Figure 10C, the protein expression of LCC is not different between the ventricles (*n* = 4; *p* > 0.05).

**FIGURE 11:**
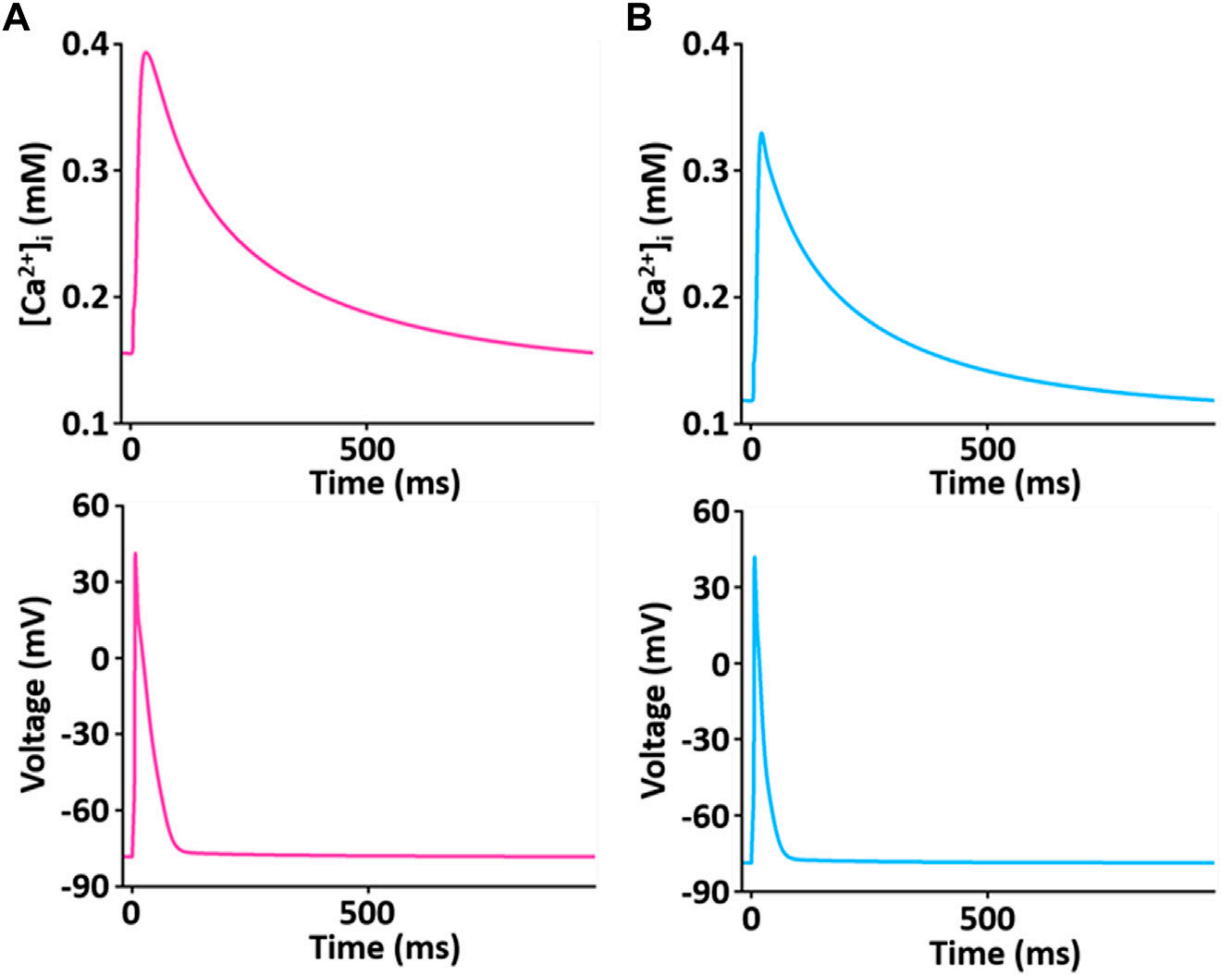
Simulated Ca^2+^ transient trace shows the underlying changes in [Ca^2+^]_i_ during corresponding AP duration for LV **(A)** and RV **(B)** myocytes at 1 Hz and 22°C (Kim, 2018).

**FIGURE 12:**
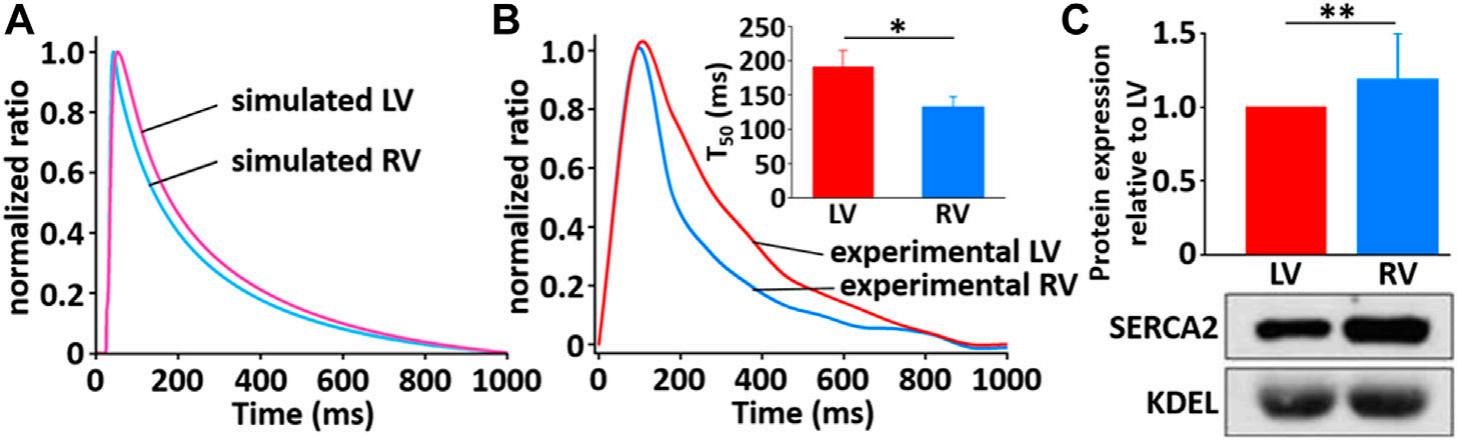
Comparison of normalized Ca^2+^ transients in model **(A)** and experimental recordings **(B)** for LV and RV myocytes at 1 Hz and 22°C (Kim, 2018). The peak ratio of the Ca^2+^ transients in the LV and RV myocytes was not different; however, the decay of the Ca^2+^ transients was much slower in LV than in RV myocyte: insert shows that T_50_ of LV cells (190.1 ms ± 24.8 ms, *n* = 6) is significantly different from that of RV cells (132.8 ms ± 15.2 ms, *n* = 6). **(C)** Verification of protein expression of SERCA2a proteins between LV and RV by immunoblotting. Representative blots and densitometric analysis of SERCA2a (100 kDa, *n* = 2) with KDEL (78 kDa) as SR marker and housekeeping protein. The bar graphs indicate mean ± SEM. *p < 0.05, **p < 0.001.

The simulated Ca^2+^ transients during the AP (Figure 11) conforms to the experimental observation that the Ca^2+^ decay is slower in LV than in RV myocytes (Figure 12), which could cause a large Ca^2+^ flux in the LV myocyte. Moreover, we assumed that the activity of SERCA in RV myocytes is higher than in LV myocytes since time to 50% decay from peak (T_50_) against [Ca^2+^]_i_ was significantly different between LV and RV myocytes (≈30%) (Figure 12B insert and *see* Taylor et al., 2004). Furthermore, as shown in Figure 12C, we revealed that SERCA2a expression of the RV is higher than that of the LV (*n* = 3; *p* < 0.001). These results can explain the underlying mechanism of the different Ca^2+^ kinetics between the LV and RV well and provide a rationale for “fine tuning” the model parameters of SERCA activity obtained from the study of Hinch et al. (2004) to reproduce a biophysical whole cell model.

### Verification of biophysical whole cell simulation: Tension production

**FIGURE 13:**
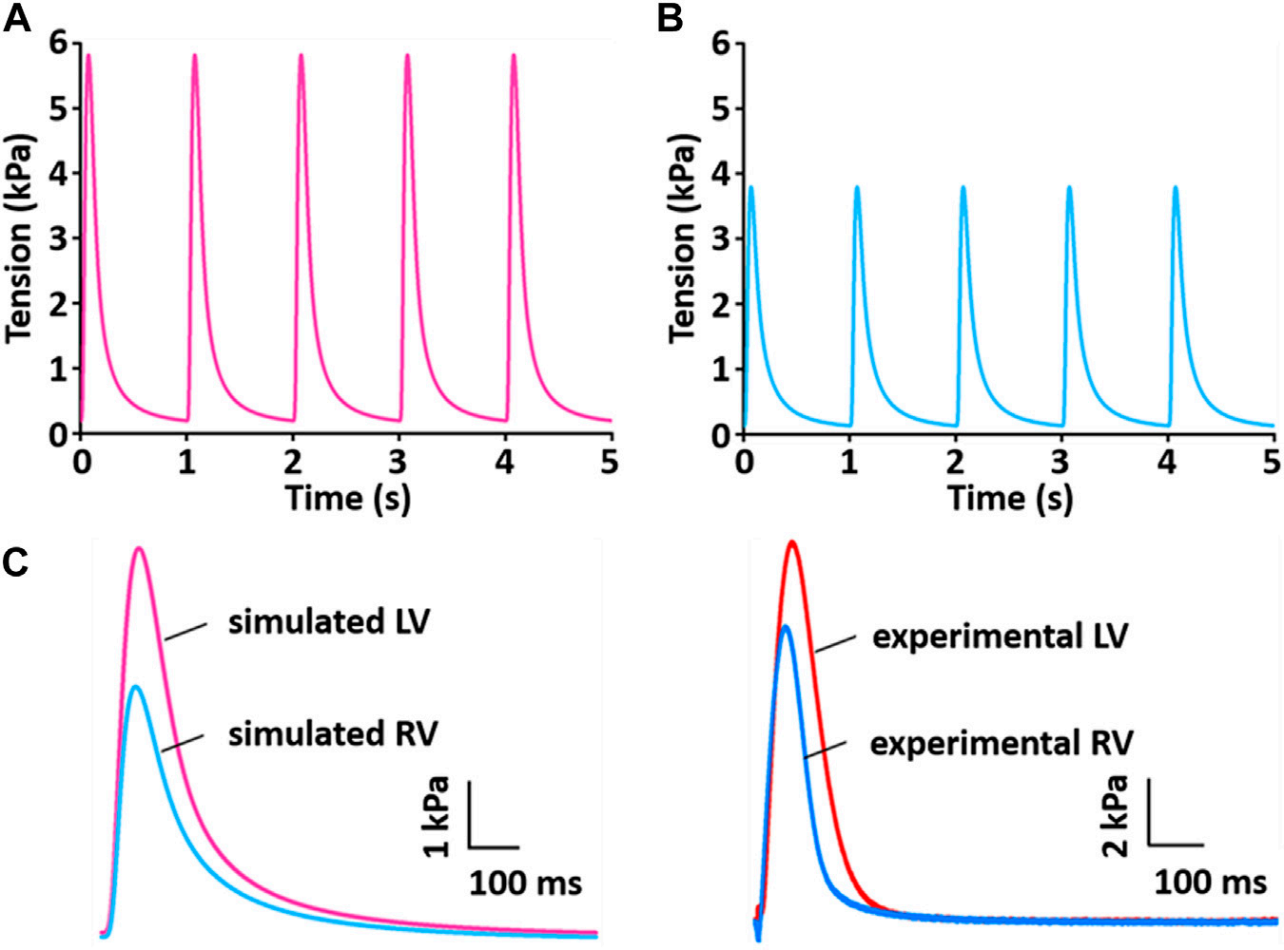
Model-generated tension production at 1 Hz and 37°C (Kim, 2018). **(A)** The tension traces for the LV model. **(B)** The tension traces for the RV model. **(C)** Comparison of simulated tension between LV and RV and validation of simulated tensions with experimentally recorded LV and RV tensions.

Figure 13 shows the simulation results for tension production over 5 s. The simulated tensions for LV and RV are in close agreement with the experimentally recorded results (Figure 13C): the peak and duration of the simulated tension are larger and longer, respectively, in the LV than in the RV myocytes. However, there is a somewhat different magnitude of generated tension between simulated and experimental results.

**FIGURE 14:**
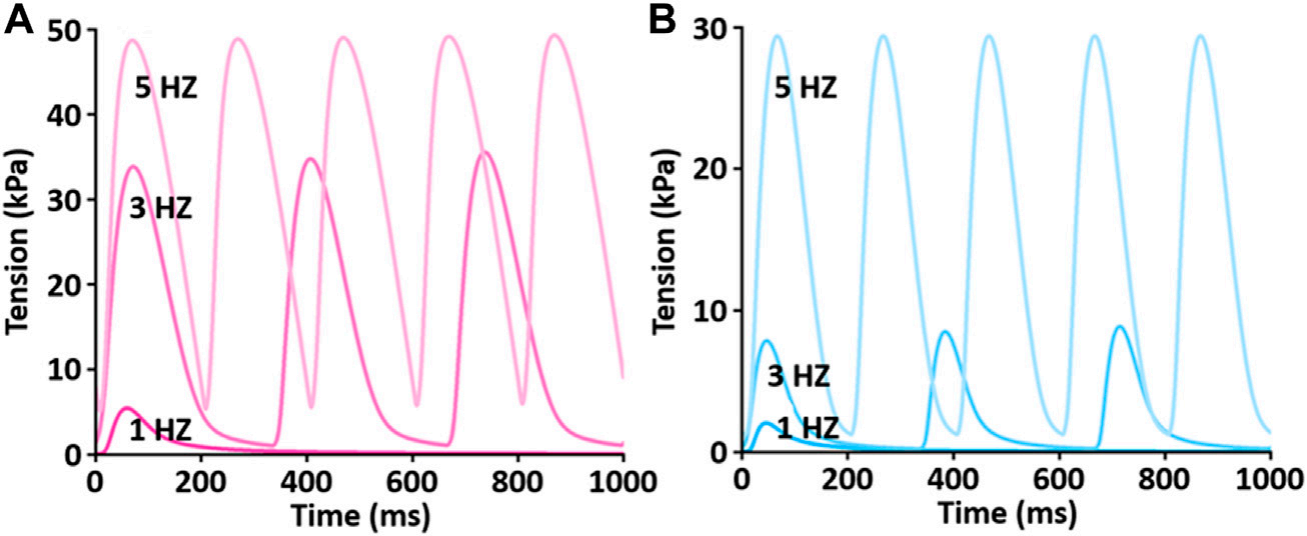
Comparison of SFR of simulated tension production between LV **(A)** and RV **(B)** myocytes at 1 Hz, 3 Hz, and 5 Hz and 37°C (Kim, 2018). Both active and passive tensions increase depending on stimulation frequency from 1 to 5 Hz.

**FIGURE 15:**
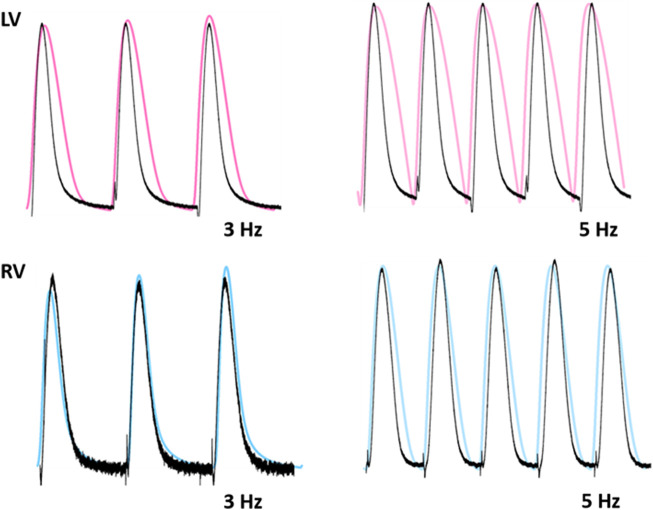
Comparison of traces for normalized tension between LV and RV myocytes at stimulations rates of 3 and 5 Hz (Kim, 2018). Black lines: experimental data. Colored lines: simulated results.

To test the functionality of the model, the model was paced at 3 and 5 Hz. The results of simulated tensions showed the expected positive stress-frequency relationship (SFR) and their kinetics are close agreement with the experimentally recorded tensions (Figures 14, 15).

## Discussion

This modeling approach serves as a good example of constructing complex models using the Physiome standard model protocol CellML in an efficient way to reuse constituents of existing computational models as modules. We assembled these reusable modules *via* component instances and encapsulation hierarchies to implement a comprehensive electromechanical model. Importantly, all modular models in this study could be subsumed into other models without modification, as each module component was constructed separately from the existing model and validated based on the results of previous related papers.

The present study is the first attempt to quantify and integrate a rat electromechanical model based on contractile proteins, as well as biophysical, experimentally derived components of ionic currents, transporters and Ca^2+^ modulators, to explain the underlying mechanisms for different stresses between LV and RV myocytes. Different AP waveforms produce different Ca^2+^ fluxes, which could be due to different SR Ca^2+^ loading and sequestration during AP. These simulation results explain why the LV exhibits greater stress compared to the RV, for which the prolonged AP duration and increased Ca^2+^ transients in the LV provide a plausible mechanism.

In this study, all simulated results of AP, LCC, Ca^2+^ transient, and tension were in close agreement with our those experimentally recorded. There is a somewhat different morphology of the APs between simulation and experiment. AP duration and shape vary from cell to cell, and may be because experimental and simulated conditions do not exactly match, or because rat AP configurations show a variation in duration and shape due to transmural heterogeneity of APs in most experiments (Stankovicova et al., 2000; Kim et al., 2010). However, it is shown that the shape of the simulated AP is very similar to experimentally recorded AP.

In adult rat ventricular myocytes, ∼ 90% of the source of the Ca^2+^ transient is Ca^2+^ released from the SR (Bers, 2002), and subsequent Ca^2+^ sequestration by SERCA matches SR release. This gives us a rationale for using SERCA as the main contributor to explain the kinetics of Ca^2+^ transients. As shown in simulations describing the changes in [Ca^2+^]_i_ (*see* also Figures 11, 12), the kinetics of Ca^2+^ transient decay in the LV myocyte are much slower than those for the RV myocyte, whereas the peak ratio of the Ca^2+^ transient is not significantly different between ventricles. This result was very well reproduced by “fine tuning” the model parameters of SERCA activity obtained from the study of Hinch et al. (2004). In fact, the decline of the Ca^2+^ transient is regulated by SERCA as well as NCX. The parameterization of NCX activity may be considered, whereas >95% of this decline is due to SERCA in rat myocytes (Milani-Nejad and Janssen, 2014). Therefore, the parameterization of SERCA activity is enough to prove the underlying mechanism for different Ca^2+^ transients between LV and RV myocytes. In addition, the underlying mechanism may be due in part to the fact that SERCA protein expression in the RV is higher than LV (Figure 12C). In the study of the force-frequency behavior of human myocardium, the protein level/activity of SERCA determines the systolic contractile reserves with respect to frequency potentiation of contractile force by playing a role in maintaining the SR load and regulating the cytosolic [Ca^2+^] during both systole and diastole (Hasenfuss et al., 1994).

Another specific aim for this simulation was to establish that the ionic mechanisms underlying the APs play a role in difference in force production between ventricles. The simulation results thus provide a good electromechanical link between the differences in Ca^2+^ handling and the corresponding tension changes. Under physiological conditions (37°C), force of contraction and speed of relaxation are complex. Thus using most variables based mainly on experimental data obtained at room temperature in the present model may limit its fidelity at 37°C, as temperature would significantly affect myofilament properties as well as Ca^2+^ handling (Janssen et al., 2002). However, even if the properties of troponin and tropomyosin and the activity of Ca^2+^ channel are adjusted, the model result is good enough to match the experimental results in this study (Figures 13–15). To verify distinct properties of contraction in the LV and RV, one of the possible ionic mechanisms, here VL of *I*_LCC_, underlying the LV and RV APs was modified, providing a good electromechanical linkage between the differences in Ca^2+^ handling and the corresponding changes in peak tension and duration of contraction.

Furthermore, Ca^2+^ transporters involved in relaxation under physiological conditions (37°C) has been reported to be accelerated compared to that at room temperature due to differences in the temperature sensitivity of the involved systems (Puglisi et al., 1996; Mackiewicz and Lewartowski, 2006). Thus, the maximum pump rate of SERCA needs to be adjusted to 37°C using the Q_10_ adjustment factor (1.415) to generate tension production as well as SFR. Among Ca^2+^ transport systems, the modification of Ca^2+^ channels and SERCA is good enough to produce different contractility between the LV and RV, which could confirm their important contributors to contractility.

### Limitations of the study and future work

In the present study, we suggested varying activity of SERCA as one of the underlying mechanisms that could contribute to the production of different stresses in the LV and RV. The activity of RYR should also be evaluated experimentally for a more comprehensive understanding of Ca^2+^ dynamics in the LV and RV. In the same context, this model is somewhat limited to Ca^2+^-dependent regulatory proteins such as CaMKII, calcineurin (CaN), phospholamban, and cAMP. These proteins alter the functions of multiple targets and thus are related to rate-dependent cellular response. LCC is influenced by Ca^2+^-dependent inactivation and Ca^2+^-dependent facilitation *via* activations of CaMKII and CaN (Pitt et al., 2001; Hudmon et al., 2005; Tandan et al., 2009). CaMKII and CaN also affect rate-dependent acceleration of relaxation *via* regulation of SERCA activity (Toyofuku et al., 1994; Munch et al., 2002). Therefore, future work is needed to implement individual modules of these proteins and then combine them with existing model.

During the diastolic phase, the myocardium stretches and passive tension is developed. Passive tension contributes to the diastolic wall tension, which determines the degree of ventricular filing and subsequent stroke volume (Allen and Kentish, 1985). At sarcomere lengths of 1.8–2.2 µm, titin and collagen are the most important contributors to passive tension (Granzier and Irving, 1995). Titin is a single giant polypeptide spanning from the Z disk to the M band region of sarcomere, with three isoforms. The isoform of titin varies with development and isoform variation causes the alteration of cardiac stiffness in the presence of heart diseases (Crocini and Gotthardt, 2021). In addition, cardiac stiffness is modulated by phosphorylation of titin by protein kinases. Therefore, in future study, it is necessary to develop a cross-bridge kinetic model that includes the role of titin.

In fact, cardiac muscle relaxation is a system-level characteristic, requiring fundamental integration of three governing systems: intracellular calcium decline, thin filament deactivation, and cross-bridge cycling kinetics (Danziger et al., 1990). Therefore, although this model is a simplified model of a complex multiphysics system, in future work we plan to use this study framework to link the overall process step by step. In addition to our modeling approach enabling the reuse of constituents from existing models, the approach used here also allows future exploration of the effects seen here at the cellular level at the tissue and organ scale. For example, tools like OpenCMISS (Bradley et al., 2011) can directly integrate CellML models, like the one developed here, into electrophysiology and mechanical models and simulations at those larger scales (Nickerson et al., 2014).

## Conclusion

In the present study, we exploit the model reuse capabilities of CellML, using individual components such as those described above. We constructed each constituent of the model separately. Once each simulation is validated, all numerical values using the CSV file were obtained. We plotted the behavior of the simulated components and justified them using experimental data. Then we implemented the comprehensive electromechanical model by assembling these reusable modules *via* component instances and encapsulation hierarchies.

In addition, our model can reproduce the experimentally observed AP, Ca^2+^ transients, and force development as a function of temperature. The model could be useful in other related experiments and simulations.

The value of the current experimental study lies in the determination of both quantitative and qualitative differences between the left and right heart at different spatial levels. Findings that the fundamental properties of the myocardium differ between the left and right heart gives us an insight on which to base medical planning or interventions, using appropriate approaches for the two ventricles. This simple approach could allow one to account for phenomenological functions which are able to capture signal traces as well as the kinetic of tension development.

The current modeling study allowed the implementation and testing of each of the currents individually, providing a database of validated modular models (component models), which are available to reuse. To construct models by reusing components encoded with CellML enables computational efficiency and easy optimization. We successfully constructed the integrated cellular model by using a modular approach with CellML. This model, informed by the present experimental data, demonstrates well how the underlying mechanisms at a molecular level contribute to phenotype in higher levels, especially based on the description of the Ca^2+^ handling mechanism in the sarcoplasmic reticulum and the sarcolemmal membrane. This enables understanding of wall stress development in the left and right ventricles. Therefore, modeling is suggested as one of the best ways to assess both left and right ventricular function together, as the assessment of right ventricular function has previously been very difficult. This approach could allow one to account for the effect of certain drugs on cardiac functions.

## Data Availability

Publicly available datasets were analyzed in this study. This data can be found here: http://hdl.handle.net/2292/36950.
