# Supplementary material for: A novel modular modeling approach for understanding different electromechanics between left and right heart in rat
Source: Front Physiol. 2022 Sep 13;13:965054. doi: 10.3389/fphys.2022.965054 (PMC9513479; doi:10.3389/fphys.2022.965054)
Supplement: Supplementary file 1 [file Table1.DOCX]

Supplementary Material

# Supplement 1

**
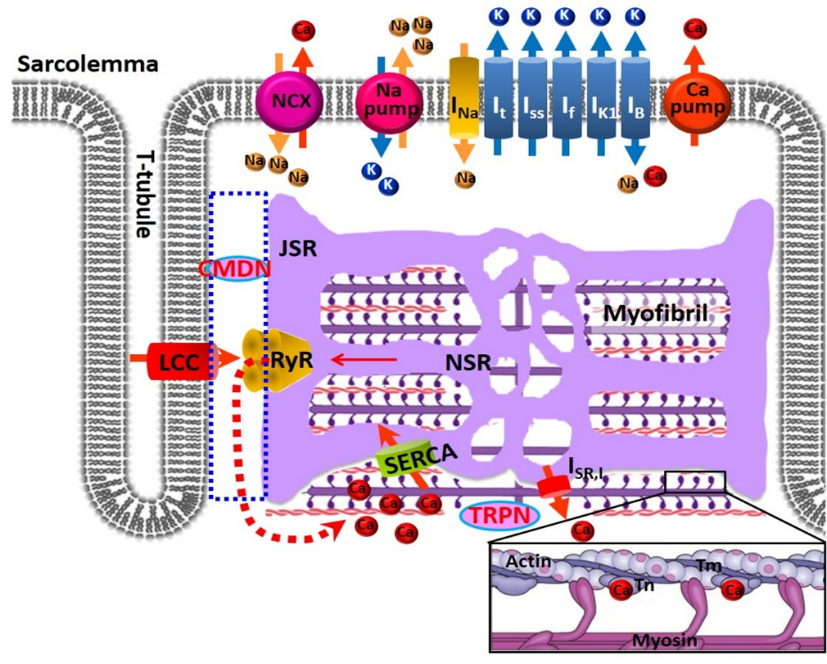
**

**Figure S1.** Schematic diagram of the currents, ion exchangers, and contractile proteins described by this study (Kim, 2018). LCC: *L*-type Ca^2+^ channel, RyR: ryanodine receptor, NCX: Na^+^-Ca^2+^ exchanger, CMDN: calmodulin, TRPN: troponin, JSR: junctional sarcoplasmic reticulum, NSR: network sarcoplasmic reticulum.


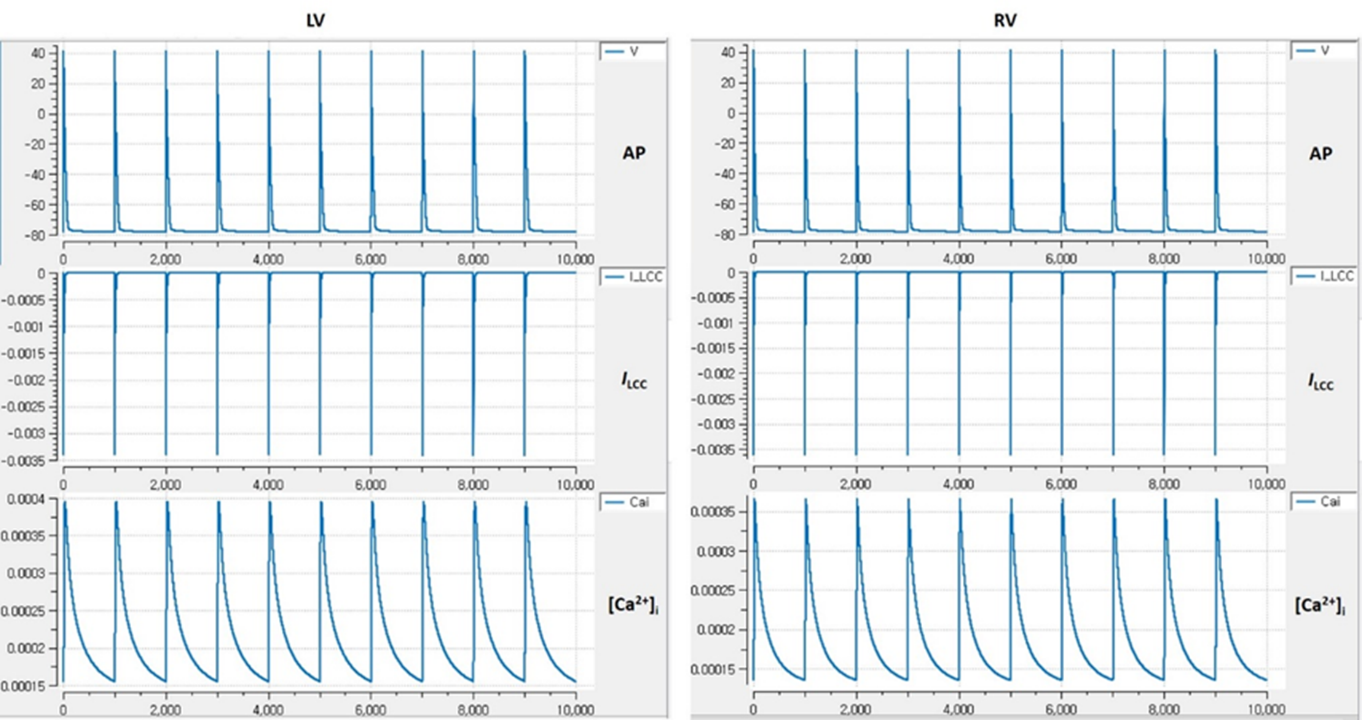


**Figure S2.** Snapshots of the resultant APs, *I*_LCC_, and calcium transients from OpenCOR for the whole cell electrophysiology model applied to LV and RV myocytes (Kim, 2018).

# The illustration of CellML text code for all units.

| **def model unit_for_model as**  **def unit millisec as**  **unit second *{pref: milli};***  **enddef;**  **def unit per_millisec as**  **unit millisec *{expo: -1};***  **enddef;**  **def unit millivolt as**  **unit volt *{pref: milli};***  **enddef;**  **def unit per_millivolt as**  **unit millivolt *{expo: -1};***  **enddef;**  **def unit millijoule_per_mole_kelvin as**  **unit joule *{pref: milli};***  **unit kelvin *{expo: -1};***  **unit mole *{expo: -1};***  **enddef;**  **def unit coulomb_per_mole as**  **unit coulomb;**  **unit mole *{expo: -1};***  **enddef;**  **def unit microA as**  **unit ampere *{pref: micro};***  **enddef;**  **def unit milliS as**  **unit siemens *{pref: milli};***  **enddef;** | **def unit per_millivolt_millisec as**  **unit per_millivolt;**  **unit per_millisec;**  **enddef;**  **def unit milliS_per_millisec as**  **unit siemens *{pref: milli};***  **unit per_millisec;**  **enddef;**  **def unit mM as**  **unit mole *{pref: milli};***  **unit litre *{expo: -1};***  **enddef;**  **def unit mM_per_millisec as**  **unit mM;**  **unit per_millisec;**  **enddef;**  **def unit mM_per_millivolt_millisec as**  **unit mM;**  **unit per_millivolt_millisec;**  **enddef;**  **def unit microL as**  **unit litre *{pref: micro};***  **enddef;**  **def unit per_mM as**  **unit mM *{expo: -1};***  **enddef;**  **def unit per_mM_millisec as**  **unit per_mM;**  **unit per_millisec;**  **enddef;** | **def unit per_millisec3 as**  **unit millisec *{expo: -3};***  **enddef;**  **def unit microm2 as**  **unit meter *{pref: micro, expo: 2};***  **enddef;**  **def unit microm3 as**  **unit meter *{pref: micro, expo: 3};***  **enddef;**  **def unit microm3_per_millisec as**  **unit microm3;**  **unit per_millisec;**  **enddef;**  **def unit microm3_mM_per_millisec as**  **unit microm3;**  **unit mM;**  **unit per_millisec;**  **enddef;**  **def unit N_per_mm2 as**  **unit newton;**  **unit metre *{pref: milli, expo: -2};***  **enddef;**  **def unit microF as**  **unit farad *{pref: micro};***  **enddef;**  **enddef;** |
| --- | --- | --- |

The illustration of CellML text code for INa module.


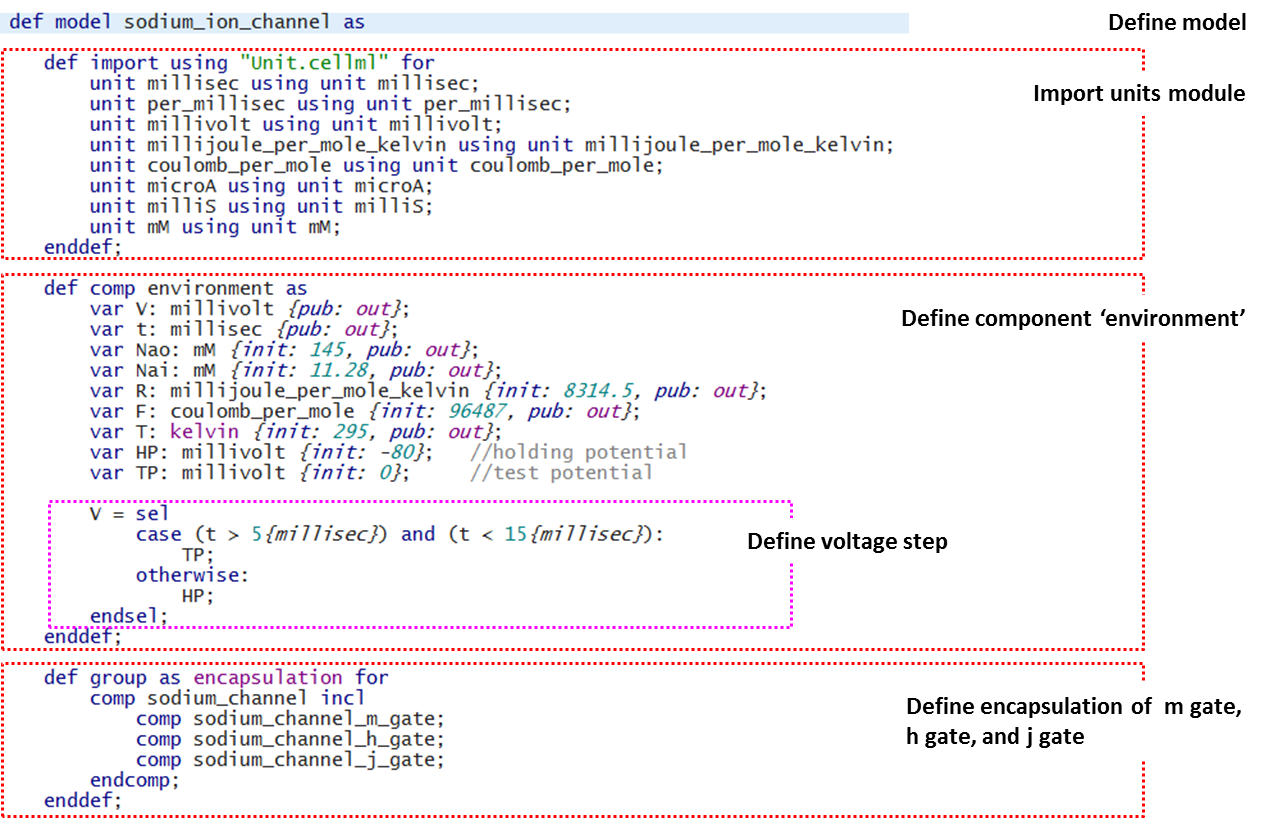


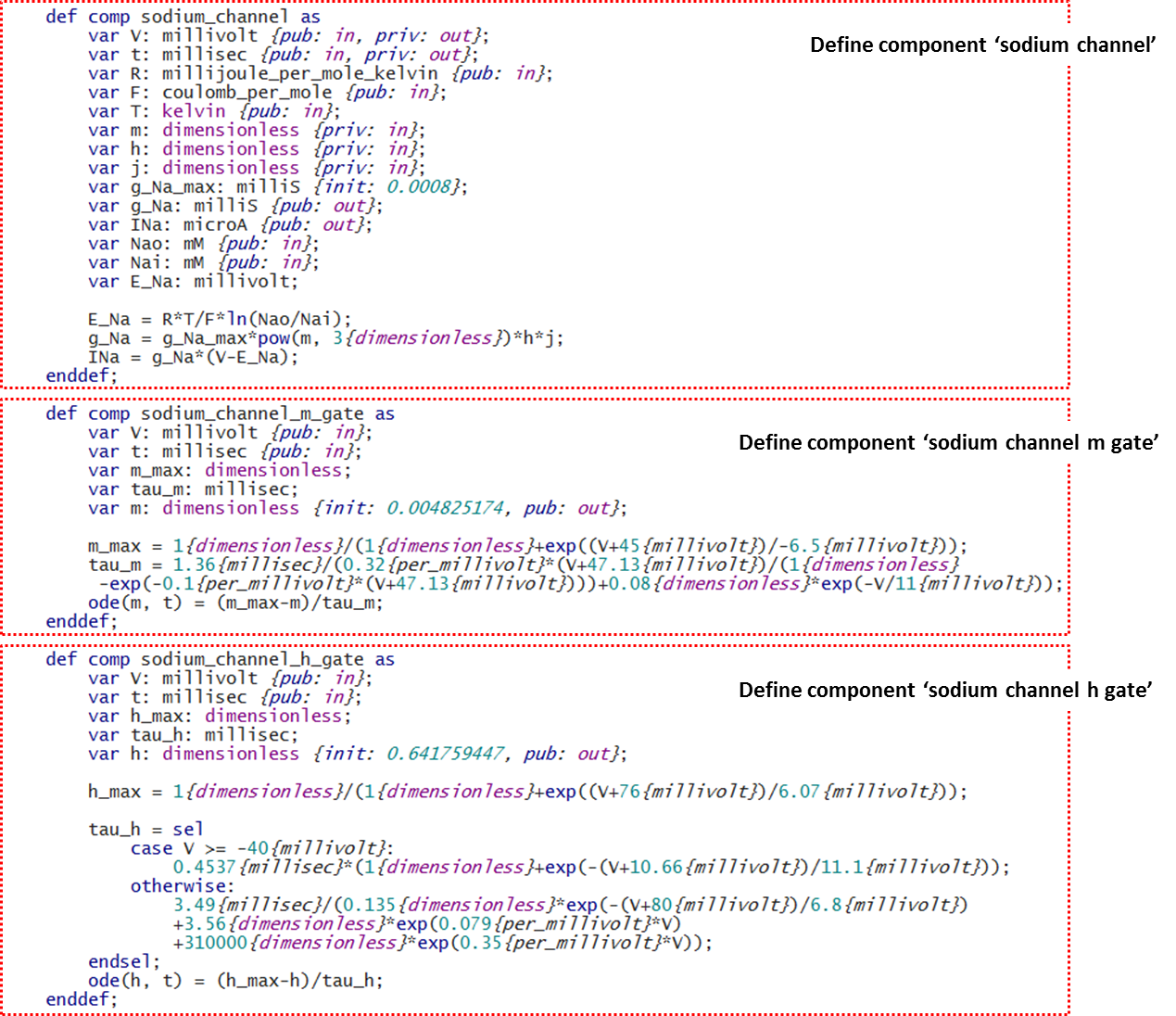


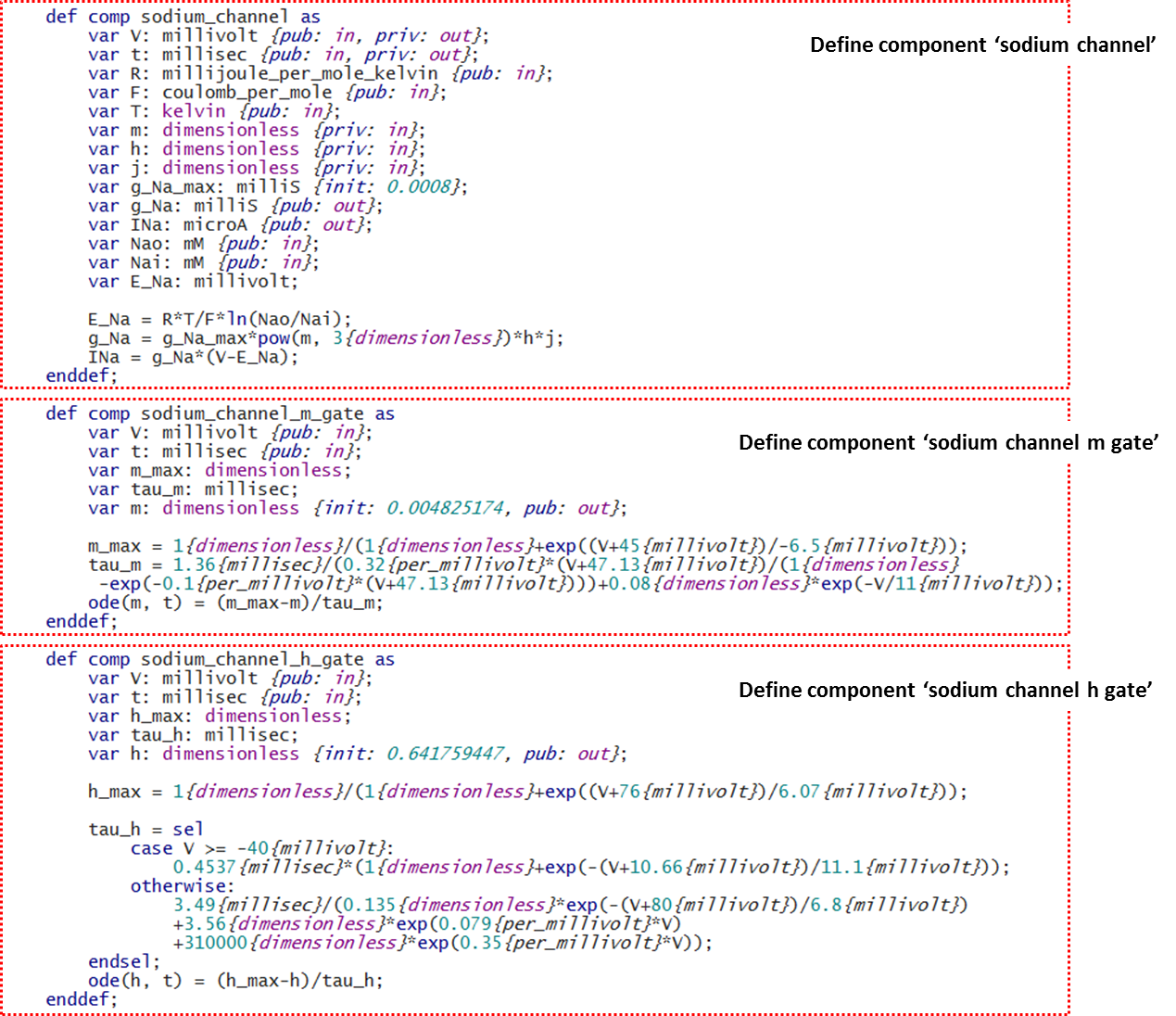


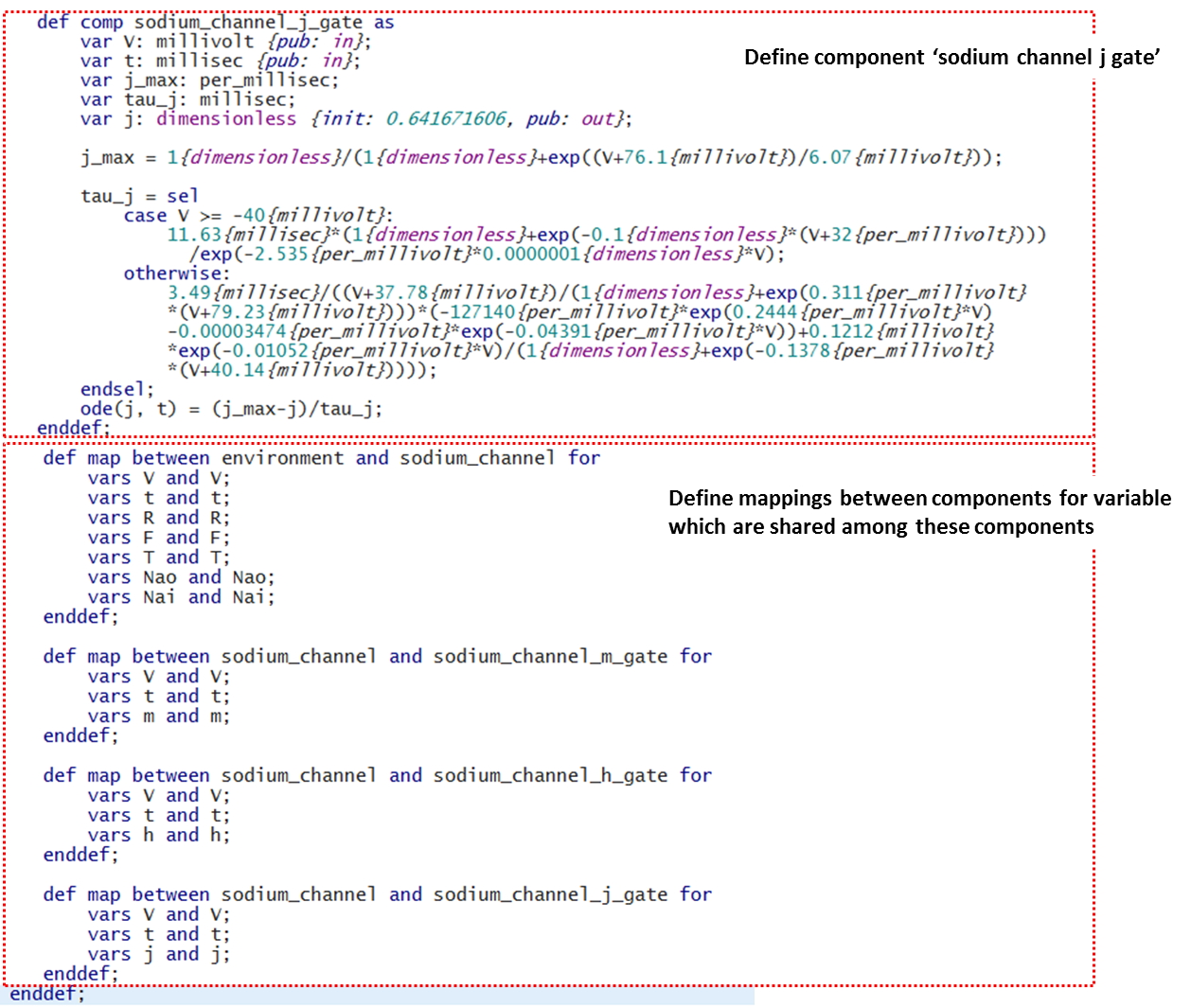


Note that several features have been addressed above: we defined components (i) the environment, (ii) the Na^+^ channel conductivity, and (iii) the dynamics of the m-, h-, and j-gate; we also defined the component maps as certain variables (V, t, R, F, T, Nao, Nai, m, h, and j) are shared between components; we added the event control select case which indicates that the voltage is specified to jump from -80 mV to 0 mV (or others) at t = 5 ms then back to -80 mV at t = 15 ms for testing the Na+ channel model; we used encapsulation to embed the sodium_channel_m_gate, h_gate, and j_gate inside the sodium_channel.

# Supplement 2: Model equations

**Membrane potential**

$$\frac{dV}{dt} = -\frac{1}{Cm}\left( I_{Na}+I_{CaL}+I_{t}+I_{ss}+I_{f}+I_{K1}+I_{B}+I_{NaK}+I_{NaCa}+I_{CaP}-I_{stim} \right)$$

**Nernst potentials**

| $E_{Na}\boldsymbol{=}\frac{RT}{F} \ln\frac{\left[ {Na}^{\boldsymbol{+}} \right]_{o}}{\left[ {Na}^{\boldsymbol{+}} \right]_{i}}$ |
| --- |

| $E_{K}\boldsymbol{=}\frac{RT}{F} \ln\frac{\left[ K^{\boldsymbol{+}} \right]_{o}}{\left[ K^{\boldsymbol{+}} \right]_{i}}$ |
| --- |

| $E_{Ca}\boldsymbol{=}\frac{RT}{2F} \ln\frac{\left[ {Ca}^{2\boldsymbol{+}} \right]_{o}}{\left[ {Ca}^{2\boldsymbol{+}} \right]_{i}}$ |
| --- |

**Na^+^ current**

| $I_{Na}=g_{Na}m^{3}hj\left( V-E_{Na} \right)$ |
| --- |

| $\bar{m} = \frac{1}{\left( 1+e^{(V+45)/-6.5} \right)}$ |
| --- |

| $\bar{h}= \bar{j}= \frac{1}{\left( 1+e^{(V+76)/6.07} \right)}$ |
| --- |

| $\frac{dm}{dt}=\frac{\bar{m}- m}{\tau_{m}}$ |  |
| --- | --- |
|  |  |
| $\frac{dh}{dt}=\frac{\bar{h}- h}{\tau_{h}}$ |  |
|  |  |
| $\frac{dj}{dt}=\frac{\bar{j}- j}{\tau_{j}}$ |  |
|  |  |
| $\tau_{m}=\frac{1.36}{\left( \frac{0.32(V+47.13)}{1.0-e^{-0.1(V+47.13)}}+0.08e^{-V/11} \right)}$ |  |
|  |  |
| $\tau_{h}=\left\{ \begin{aligned} \frac{3.49}{0.135e^{-\frac{V+80}{6.8}}+3.5e^{0.079V}+3.1\times{10}^{5}e^{0.35V}}, &V<-40mV \\ 0.0004537\left( 1.0+e^{-\frac{\left( V+10.66 \right)}{11.1}} \right) , &V\geq-40 mV \end{aligned} \right.$ | |

$$\tau_{j}=\left\{ \begin{aligned} 3.49 \div\left[ \begin{aligned} \frac{V+37.78}{1.0+e^{0.311\left( V+79.23 \right)}}\left( -127140e^{0.2444V}-3.474\times{10}^{-5}e^{-0.04391V} \right) \\ +\frac{0.1212e^{-0.01052V}}{1.0+e^{-0.1378\left( V+40.14 \right)}} \end{aligned} \right], & \\ V<-40mV \\ \\ \frac{11.63\left( 1.0+e^{-\frac{\left( V+32 \right)}{11.1}} \right)}{e^{-2.535\times{10}^{-7}V}} , & \\ V\geq-40 mV \end{aligned} \right.$$

**Ca^2+^-independent transient outward K^+^ current**

| $I_{t}=g_{t}r(as+bs_{slow})\left( V-E_{K} \right)$ |
| --- |
|  |

| $a=0.886, b=0.114$ | |
| --- | --- |
|  | |
| $\bar{r} = \frac{1}{\left( 1+e^{(V+10.6)/-11.42} \right)}$ |  |
|  |  |
| $\bar{s}= \bar{s_{slow}}= \frac{1}{\left( 1+e^{(V+45.3)/6.8841} \right)}$ |  |
|  |  |
| $\tau_{r}=\frac{1000}{{45.16e}^{0.03577(V+50.0)}+98.9e^{-0.1(V+38.0)}}$ |  |
|  |  |
| $\tau_{s}={350e}^{-{(V+70.0/15.0)}^{2}}+35$ |  |
|  |  |
| $\tau_{s_{slow}}={3700e}^{-{(V+70.0/30.0)}^{2}}+35$ |  |
|  |  |
| $\frac{dr}{dt}=\frac{\bar{r}- r}{\tau_{r}}$ |  |
|  |  |
| $\frac{ds}{dt}=\frac{\bar{s}- s}{\tau_{s}}$ |  |
|  |  |
| $\frac{ds_{slow}}{dt}=\frac{\bar{s_{slow}}- s_{slow}}{\tau_{s_{slow}}}$ |  |

**Steady-state outward K^+^ current**

| $I_{ss}=g_{ss}r_{ss}s_{ss}\left( V-E_{K} \right)$ |
| --- |
|  |
| $\bar{r_{ss}} = \frac{1}{\left. 1+e^{(V+11.5)/-11.82} \right.}$ |

| $\bar{s_{ss}}= \frac{1}{\left. 1+e^{(V+87.5)/10.3} \right.}$ |
| --- |
|  |
| $\tau_{r_{ss}}=\frac{10000}{{45.16e}^{0.03577(V+50.0)}+98.9e^{-0.1(V+38.0)}}$ |
|  |

$\tau_{s_{ss}}=2100$

| $\frac{dr_{ss}}{dt}=\frac{\bar{r_{ss}}- r_{ss}}{\tau_{r_{ss}}}$ |
| --- |
|  |
| $\frac{ds_{ss}}{dt}=\frac{\bar{s_{ss}}- s_{ss}}{\tau_{s_{ss}}}$ |

**Inward rectifier K^+^ current**

| $I_{K1}=\left[ \frac{0.048}{e^{\frac{\left( V+37 \right)}{25}}+e^{\frac{\left( V+37 \right)}{-25}}}+0.01 \right]\cdot\left[ \frac{0.001}{1+e^{\frac{\left( V-E_{K}-76.77 \right)}{-17}}} \right]+\frac{g_{K1}(V-E_{K}-1.73)}{{(1+e}^{1.613F\left( V-E_{K}-1.73 \right)/RT})\cdot(1+e^{\left[ K^{\boldsymbol{+}} \right]_{o}-0.9988/-0.124})}$ |
| --- |

**Hyperpolarizing-activated current**

| $I_{f}=g_{f}y[f_{Na}\left( V-E_{Na} \right)+f_{K}\left( V-E_{K} \right)]$ |
| --- |
|  |
| $y_{\infty} = \frac{1}{\left. 1+e^{(V+138.6)/10.48} \right.}$ |
|  |

$$f_{Na}=0.2, f_{K}=1-f_{Na}$$

| $\tau_{y}=\frac{1000}{{(0.11885e}^{(V+80.0)/28.37}+0.56236e^{(V+80.0)/-14.19})}$ |
| --- |
|  |
| $\frac{dy}{dt}=\frac{\bar{y_{\infty}}- y}{\tau_{y}}$ |

**Background Na^+^ current**

| $I_{BNa}=g_{BNa}\left( V-E_{Na} \right)$ |
| --- |

**Background K^+^ current**

| $I_{BK}=g_{BK}\left( V-E_{K} \right)$ |
| --- |

**Na^+^-K^+^ pump current**

| $I_{NaK}=\frac{\left( \bar{I_{NaK}}\cdot\frac{1}{{(1.0+ 0.1245e}^{-\frac{0.1VF}{RT}}+0.0365e^{\frac{VF}{RT}})} \cdot\frac{\left[ K^{\boldsymbol{+}} \right]_{o}}{\left( \left[ K^{\boldsymbol{+}} \right]_{o}+k_{m,k} \right)} \right)}{\left( 1+ \frac{k_{m,Na}}{\left. \left[ {Na}^{\boldsymbol{+}} \right]_{i} \right.} \right)^{4}}$ |
| --- |

**Intracellular ion concentration**

| $\frac{d\left[ {Na}^{\boldsymbol{+}} \right]_{i}}{dt}= -(I_{Na}+I_{BNa}+3I_{NaCa}+3I_{NaK}+I_{f,Na})\frac{1.0}{V_{myo\_\mu l}F}$ |
| --- |
|  |
| $\frac{d\left[ K^{\boldsymbol{+}} \right]_{i}}{dt}= -(I_{ss}+I_{BK}+I_{t}+I_{K1}+I_{f,K}-2I_{NaK})\frac{1.0}{V_{myo\_\mu l}F}$ |

**Calcium dynamics**

***Calcium concentration***

| $\frac{d\left[ {Ca}^{2\boldsymbol{+}} \right]_{i}}{dt}=\beta_{i} \left\{ I_{RyR}-I_{SERCA}+I_{SR}+I_{TRPN}-\left( {I_{LCC}+I}_{BCa}-2I_{NaCa}+I_{CaP} \right)\frac{1.0}{{2V}_{myo}F} \right\}$ |
| --- |
|  |
| $\frac{d\left[ {Ca}^{2\boldsymbol{+}} \right]_{SR}}{dt}=\left( -{I_{RyR}+I}_{SERCA}-I_{SR,l} \right)\frac{V_{myo\_\mu l}}{V_{SR\_\mu l}}$ |
|  |
| $\frac{d[TRPN]}{dt}=I_{TRPN}$ |

***Dyadic space calcium concentrations***

| $C_{cc}={[{Ca}^{2+}]}_{i}$ |
| --- |
|  |
| $C_{co}=\frac{{[{Ca}^{2+}]}_{i}+\frac{J_{R}}{g_{D}}{[{Ca}^{2+}]}_{SR}}{1+\frac{J_{R}}{g_{D}}}$ |
|  |
| $C_{oc}=\frac{{[{Ca}^{2+}]}_{i}+\frac{J_{L}}{g_{D}}{[{Ca}^{2+}]}_{e}\frac{\delta Ve^{-\delta V}}{1-e^{-\delta V}}}{1+\frac{J_{R}}{g_{D}}\frac{\delta V}{1-e^{-\delta V}}}$ |
|  |
| $C_{oo}=\frac{{[{Ca}^{2+}]}_{i}+\frac{J_{R}}{g_{D}}{[{Ca}^{2+}]}_{SR}+\frac{J_{L}}{g_{D}}{[{Ca}^{2+}]}_{e}\frac{\delta Ve^{-\delta V}}{1-e^{-\delta V}}}{1+\frac{J_{R}}{g_{D}}+\frac{J_{L}}{g_{D}}\frac{\delta V}{1-e^{-\delta V}}}$ |

***Transition rates for the three-state LCC***

| $\alpha^{+}=\frac{e^{\frac{(V-V_{L})}{\Delta V_{L}}}}{t_{L}\left( e^{\frac{\left( V-V_{L} \right)}{\Delta V_{L}}}+ 1 \right)}$ | |
| --- | --- |
|  | |
| $\alpha^{-}=\frac{\emptyset_{L}}{t_{L}}$ | |
|  | |
| $\epsilon_{co}^{+}=\frac{C_{co}\left( e^{\frac{\left( V-V_{L} \right)}{\Delta V_{L}}}+ a \right)}{\tau_{L}K_{L}\left( e^{\frac{\left( V-V_{L} \right)}{\Delta V_{L}}}+ 1 \right)}$ |  |
|  |  |
| $\epsilon_{cc}^{+}=\frac{{[{Ca}^{2+}]}_{i}\left( e^{\frac{\left( V-V_{L} \right)}{\Delta V_{L}}}+ a \right)}{\tau_{L}K_{L}\left( e^{\frac{\left( V-V_{L} \right)}{\Delta V_{L}}}+ 1 \right)}$ |  |
|  |  |
| $\epsilon^{-}=\frac{b\left( e^{\frac{\left( V-V_{L} \right)}{\Delta V_{L}}}+ a \right)}{\tau_{L}\left( {be}^{\frac{\left( V-V_{L} \right)}{\Delta V_{L}}}+ a \right)}$ |  |

***Transition rates for the three-state RYR***

| $\beta_{oc}^{+}=\frac{\left( C_{oc} \right)^{2}}{t_{R}\left( \left( C_{oc} \right)^{2}+ \left( K_{RyR} \right)^{2} \right)}$ |
| --- |
|  |
| $\beta_{cc}^{+}=\frac{\left( {[{Ca}^{2+}]}_{i} \right)^{2}}{t_{R}\left( \left( {[{Ca}^{2+}]}_{i} \right)^{2}+ \left( K_{RyR} \right)^{2} \right)}$ |
|  |
| $\beta^{-}=\frac{\emptyset_{R}}{t_{R}}$ |
|  |
| $\mu_{oc}^{+}=\frac{\left( C_{oc} \right)^{2}+{c\left( K_{RyR} \right)}^{2}}{\tau_{R}\left( \left( C_{oc} \right)^{2}+ \left( K_{RyR} \right)^{2} \right)}$ |
|  |
| $\mu_{cc}^{+}=\frac{\left( {[{Ca}^{2+}]}_{i} \right)^{2}+{c\left( K_{RyR} \right)}^{2}}{\tau_{R}\left( \left( {[{Ca}^{2+}]}_{i} \right)^{2}+ \left( K_{RyR} \right)^{2} \right)}$ |
|  |
| $\mu_{oc}^{-}=\frac{\theta_{R}d\left( \left( C_{oc} \right)^{2}+{c\left( K_{RyR} \right)}^{2} \right)}{\tau_{R}\left( {d\left( C_{oc} \right)}^{2}+ {c\left( K_{RyR} \right)}^{2} \right)}$ |
|  |
| $\mu_{cc}^{-}=\frac{\theta_{R}d\left( \left( {[{Ca}^{2+}]}_{i} \right)^{2}+{c\left( K_{RyR} \right)}^{2} \right)}{\tau_{R}\left( {d\left( {[{Ca}^{2+}]}_{i} \right)}^{2}+ {c\left( K_{RyR} \right)}^{2} \right)}$ |

***Conditional probabilities of the combined states of the calcium release unit***

| \| $P\left( y_{oc} \vert z_{1} \right)=\frac{\alpha^{+}\beta^{-}(\alpha^{+}+\alpha^{-}+\beta^{-}+\beta_{cc}^{+})}{\left( \alpha^{+}+\alpha^{-} \right)(\left( \alpha^{-}+\beta^{-}+\beta_{oc}^{+} \right)\left( \beta^{-}+\beta_{cc}^{+} \right)+\alpha^{+}\left( \beta^{-}+\beta_{oc}^{+} \right))}$ \| \| --- \|      \| $P\left( y_{co} \vert z_{1} \right)=\frac{\alpha^{+}\beta_{cc}^{+}\left( \alpha^{-}+\beta^{-}+\beta_{oc}^{+} \right)+\beta_{oc}^{+}\alpha^{+}}{\left( \alpha^{+}+\alpha^{-} \right)(\left( \alpha^{-}+\beta^{-}+\beta_{oc}^{+} \right)\left( \beta^{-}+\beta_{cc}^{+} \right)+\alpha^{+}\left( \beta^{-}+\beta_{oc}^{+} \right))}$ \| \| --- \|  \| $P\left( y_{oo} \vert z_{1} \right)=\frac{\alpha^{+}\beta_{oc}^{+}\left( \alpha^{+}+\beta^{-}+\beta_{cc}^{+} \right)+\beta_{cc}^{+}\alpha^{-}}{\left( \alpha^{+}+\alpha^{-} \right)(\left( \alpha^{-}+\beta^{-}+\beta_{oc}^{+} \right)\left( \beta^{-}+\beta_{cc}^{+} \right)+\alpha^{+}\left( \beta^{-}+\beta_{oc}^{+} \right))}$ \| \| --- \|  \| $P\left( y_{cc} \vert z_{1} \right)=\frac{\alpha^{-}\beta^{-}\left( \alpha^{+}+\alpha^{-}+\beta^{-}+\beta_{oc}^{+} \right)}{\left( \alpha^{+}+\alpha^{-} \right)(\left( \alpha^{-}+\beta^{-}+\beta_{oc}^{+} \right)\left( \beta^{-}+\beta_{cc}^{+} \right)+\alpha^{+}\left( \beta^{-}+\beta_{oc}^{+} \right))}$ \| \| --- \|  \| $P\left( y_{oi} \vert z_{2} \right)=\frac{\alpha^{+}}{\alpha^{+}+\alpha^{-}}$ \| \| --- \|  \| $P\left( y_{ci} \vert z_{2} \right)=\frac{\alpha^{-}}{\alpha^{+}+\alpha^{-}}$ \| \| --- \|  \| $P\left( y_{ic} \vert z_{3} \right)=\frac{\beta^{-}}{\beta_{cc}^{+}+\beta^{-}}$ \| \| --- \|  \| $P\left( y_{io} \vert z_{3} \right)=\frac{\beta_{cc}^{+}}{\beta_{cc}^{+}+\beta^{-}}$ \| \| --- \|  \| $P\left( y_{io} \vert z_{3} \right)=\frac{\beta_{cc}^{+}}{\beta_{cc}^{+}+\beta^{-}}$ \| \| --- \| |
| --- | --- | --- | --- | --- | --- | --- | --- | --- | --- |

***Transition rates between the combined states of the calcium release unit***

| $r_{1}=P\left( y_{oc} \vert z_{1} \right)\mu_{oc}^{+}+P\left( y_{cc} \vert z_{1} \right)\mu_{cc}^{+}$ |
| --- |
|  |
| $r_{2}=\frac{{\alpha^{+}\mu}_{oc}^{-}+{\alpha^{-}\mu}_{cc}^{-}}{\alpha^{+}+\alpha^{-}}$ |
|  |
| $r_{3}=\frac{{\beta^{-}\mu}_{cc}^{+}}{\beta_{cc}^{+}+\beta^{-}}$ |
|  |
| $r_{4}=\mu_{cc}^{-}$ |

| $r_{5}=P\left( y_{co} \vert z_{1} \right)\epsilon_{co}^{+}+P\left( y_{cc} \vert z_{1} \right)\epsilon_{cc}^{+}$ |
| --- |
|  |
| $r_{6}=\epsilon^{-}$ |
|  |
| $r_{7}=\frac{{\alpha^{-}\epsilon}_{cc}^{+}}{\alpha^{+}+\alpha^{-}}$ |
|  |
| $r_{8}=\epsilon^{-}$ |

***Calcium fluxes***

| $J_{R,co}=J_{R}\frac{{[{Ca}^{2+}]}_{SR}-{[{Ca}^{2+}]}_{i}}{1+\frac{J_{R}}{g_{D}}}$ |  |  |
| --- | --- | --- |
|  |  |  |
| $J_{R,oo}=J_{R}\frac{\left( {[{Ca}^{2+}]}_{SR}-{[{Ca}^{2+}]}_{i}+\frac{J_{L}}{g_{D}}\cdot\frac{\delta V}{1-e^{-\delta V}}({[{Ca}^{2+}]}_{SR}-{[{Ca}^{2+}]}_{e}e^{-\delta V}) \right)}{1+\frac{J_{R}}{g_{D}}+\frac{J_{L}}{g_{D}}\cdot\frac{\delta V}{1-e^{-\delta V}}}$ | |  |
|  | |  |
| $J_{L,oc}=J_{L}\frac{\delta V}{1-e^{-\delta V}}\cdot\frac{\left( {[{Ca}^{2+}]}_{e}e^{-\delta V}-{[{Ca}^{2+}]}_{i} \right)}{1+\frac{J_{L}}{g_{D}}\cdot\frac{\delta V}{1-e^{-\delta V}}}$ |  |  |
|  |  |  |
| $J_{L,oo}=J_{L}\frac{\delta V}{1-e^{-\delta V}}\cdot\frac{\left( {[{Ca}^{2+}]}_{e}e^{-\delta V}-\left[ {Ca}^{2+} \right]_{i}+\frac{J_{R}}{g_{D}}({[{Ca}^{2+}]}_{e}e^{-\delta V}-{[{Ca}^{2+}]}_{SR}) \right)}{1+\frac{J_{R}}{g_{D}}+\frac{J_{L}}{g_{D}}\frac{\delta V}{1-e^{-\delta V}}}$ | | |

***Calcium buffering***

| $I_{TRPN}=2V_{myo}F\left\{ {k^{-}}_{TRPN}\left( {[B]}_{TRPN}-\left[ TRPN \right] \right)-{k^{+}}_{TRPN}[TRPN]{[{Ca}^{2+}]}_{i} \right\}$ | |
| --- | --- |
|  | |
| $\beta\_CMDN=\left( 1+\frac{K_{CMDN}{[B]}_{CMDN}}{\left( K_{CMDN}+{[{Ca}^{2+}]}_{i} \right)^{2}}+\frac{K_{m}^{EGTA}{[EGTA]}_{tot}}{\left( K_{m}^{EGTA}+{[{Ca}^{2+}]}_{i} \right)^{2}} \right)^{-1}$ |  |

***Calcium release unit***

| $\frac{dz_{1}}{dt}=-\left( r_{1}+r_{5} \right)z_{1}+r_{2}z_{2}+r_{6}z_{3}$ |  |
| --- | --- |
|  |  |
| $\frac{dz_{2}}{dt}=r_{1}z_{1}-\left( r_{2}+r_{7} \right)z_{2}+r_{8}(1-z_{1}-z_{2}-z_{3})$ | |

| $\frac{dz_{3}}{dt}=r_{5}z_{1}-\left( r_{6}+r_{3} \right)z_{3}+r_{4}(1-z_{1}-z_{2}-z_{3})$ |
| --- |

***L-type Ca^2+^ current***

| $I_{LCC}=\frac{N}{V_{myo}}\left( {(J}_{L,oo}P\left( y_{oo} \vert z_{1} \right)+J_{L,oc}P\left( y_{oc} \vert z_{1} \right))z_{1}+\frac{J_{L,oc}\alpha^{+}}{\alpha^{+}+\alpha^{-}}z_{2} \right)(-2\times V_{myo\_\mu l} \times F)$ |
| --- |

***Ryanodine receptor***

| $I_{RyR}=\frac{N}{V_{myo}}\left( {(J}_{R,oo}P\left( y_{oo} \vert z_{1} \right)+J_{R,co}P\left( y_{co} \vert z_{1} \right))z_{1}+\frac{J_{R,co}\beta_{cc}^{+}}{\beta_{cc}^{+}+\beta^{-}}z_{3} \right)$ |
| --- |

**SR Ca^2+^ pump current**

| $I_{SERCA}=g_{SERCA}\frac{{[{Ca}^{2+}]}_{i}^{2}}{K_{SERCA}^{2}+{[{Ca}^{2+}]}_{i}^{2}}$ |
| --- |

**Sarcoplasmic Ca^2+^ pump current**

| $I_{pCa}=\frac{g_{pCa}{[\mathrm{Ca}^{2+}]}_{i}}{{[\mathrm{Ca}^{2+}]}_{i}+K_{m,pCa}} (2\times V_{myo\_\mu l}\times F)$ |
| --- |

**Na^+^-Ca^2+^ exchanger current**

| $I_{NCX}=g_{NCX}\frac{e^{VF/RT}{[{Na}^{+}]}_{i}^{3}{[{Ca}^{2+}]}_{e}-e^{(-1)VF/RT}{[{Na}^{+}]}_{e}^{3}{[{Ca}^{2+}]}_{i}}{\left( K_{m,Na}^{3}+\left[ {Na}^{+} \right]_{e}^{3} \right)\left( \left[ {Ca}^{2+} \right]_{e}+K_{m,Ca} \right)(1+k_{sat}e^{(-1)VF/RT})}$ |
| --- |

**Background Ca^2+^ current**

| $I_{BCa}=g_{BCa}\left( V-E_{Ca} \right)(-2\times V_{myo\_\mu l}\times F)$ |
| --- |

**Troponin**

$$I_{TRPN}=\left. k_{on}\left( {[{Ca}^{2+}]}_{TRPN\_max}-\left[ TRPN \right] \right)-k_{off}[TRPN]{[{Ca}^{2+}]}_{i} \right.$$

**Tropomyosin**

$$\frac{dz}{dt}=\alpha_{0}\left( \frac{{[{Ca}^{2+}]}_{Trpn}}{{[{Ca}^{2+}]}_{Trpn50}} \right)^{n}\left( 1-z \right)-\alpha_{r1}z-\alpha_{r2}\frac{z^{n_{r}}}{z^{n_{r}}+K_{z}^{n_{r}}}$$

**Crossbridge**

$$T=\left\{ \begin{aligned} T_{0}\frac{a\sum_{i=1}^{n} Q_{i}+1}{1 - \sum_{i=1}^{n} Q_{i}}, &\sum_{i=1}^{n} Q_{i} <0 \\ T_{0}\frac{1 +(2+a)\sum_{i=1}^{n} Q_{i}}{1 + \sum_{i=1}^{n} Q_{i}}, &\sum_{i=1}^{n} Q_{i} >0 \end{aligned} \right.$$

$$T= T_{ref}\times(1+\beta_{0}(\lambda-1)) \times\frac{z}{z_{Max}}$$

**Table S1. Physical constants and geometry**

| **Parameter** | **Definition** | **Value** |
| --- | --- | --- |
| **F** | Faraday's constant | 96.487 kC·M^-1^ |
| **T** | Temperature | 295 K |
| **R** | Universal gas constant | 8.3145 J·M^-1^·K^-1^ |
| **V_myo_** | Volume of myoplasm | 25850 μm^3^ |
| **V_SR_** | Volume of SR | 2098 μm^3^ |
| **V_myo_μl_** |  | 25.85 pL |
| **V_SR_μl_** |  | 2.098 pL |
| **[Na^+^]_o_** | Extracellular Na^+^ concentration | 145 mM·L^-1^ |
| **[K^+^]_o_** | Intracellular K^+^ concentration | 5.4 mM·L^-1^ |
| **[Ca^2+^]_o_** | Extracellular Ca^2+^ concentration | 1.2 mM·L^-1^ |

**Table S2. Membrane current parameters**

| **Parameter** | **Definition** | **Value** |
| --- | --- | --- |
| **Cm** | Total membrane capacitance | 100 pF |
| **g_Na_** | Maximum conductance for I_Na_ | 0.8 µS |
| **g_t_** | Maximum conductance for I_t_ | 17.5 nS |
| **g_ss_** | Maximum conductance for I_ss_ | 0.7 nS |
| **g_K1_** | Maximum conductance for I_K1_ | 24 nS |
| **g_BNa_** | Maximum conductance for I_BNa_ | 80.15 pS |
| **g_BK_** | Maximum conductance for I_BK_ | 138 pS |
| **g_f_** | Maximum conductance for I_f_ | 145 pS |
| **I_NaK,max_** | Maximum I_NaK_ | 95 pA |
| **K_m,Na_** | Half-maximum Na^+^ binding constant for I_NaK_ | 10 M·L^-1^ |
| **K_m,K_** | Half-maximum K^+^ binding constant for I_NaK_ | 1.5 M·L^-1^ |

**Table S3. Ca^2+^ handling parameters**

| **Parameter** | **Definition** | **Value** |
| --- | --- | --- |
| **g_D_** | Calcium flux rate from dyadic space to cytosol | 65 μm^3^·s^-1^ |
| **J_R_** | Permeability of single RYR | 20 μm^3^·s^-1^ |
| **J_L_** | Permeability of single LCC | 0.913 μm^3^·s^-1^ |
| **N** | Number of release units | 50000 |
| **VL** | Potential when half LCC open | -15 mV |
| **ΔV_L_** | Width of opening potentials | 7 mV |
| **φ_L_** | Proportion of time closed in open mode | 2.35 |
| **t_L_** | Time switching between C and O states | 1 ms |
| **t_R_** | Time switching between C and O states | 1.17 x t_L ms |
| **τ_L_** | Inactivation time | 650 ms |
| **τ_R_** | Inactivation time | 2.43 ms |
| **φ_R_** | Proportion of time closed in open mode | 0.05 |
| **θ_R_** | Reciprocal of proportion of time inactivated in open mode | 0.012 |
| **K_RyR_** | Half concentration of activation | 41 μM·L^-1^ |
| **K_L_** | Concentration at inactivation | 0.22 μM·L^-1^ |
| **a** |  | 0.0625 |
| **b** |  | 14 |
| **c** | Biasing to make inactivation a function of [Ca^2+^]_ds_ | 0.01 |
| **d** | Biasing to make inactivation a function of [Ca^2+^]_ds_ | 100 |
| **K_mNa_** | Sodium half saturation of NCX | 87.5 mM·L^-1^ |
| **K_mCa_** | Calcium half saturation of NCX | 1.38 mM·L^-1^ |
| **η** | Voltage dependence of NCX control | 0.35 |
| **k_sat_** | Low potential saturation factor of NCX | 0.1 |
| **g_NCX_** | Pump rate of NCX | 38.5 μM·L^-1^·s^-1^ |
| **g_SERCA_** | Maximum pump rate of SERCA | 0.45 μM·L^-1^·s^-1^ |
| **K_SERCA_** | Half saturation of SERCA | 0.5 μM·L^-1^ |
| **g_pCa_** | Maximum I_pCa_ | 0.7 μS·s^-1^ |
| **K_pCa_** | Half saturation of sarcolemmal pump | 0.5 μM·L^-1^ |
| **g_BCa_** | Maximum conductance for I_BCa_ | 26.875 μM·L^-1^·V^-1^·s^-1^ |
| **g_SR,l_** | Rate of leak from SR to cytosol | 0.018951 s^-1^ |
| **[B]_CMDN_** | Total cytosolic calmodulin concentration | 0.05 mM·L^-1^ |
| **[K]_CMDN_** | Half saturation constant of calmodulin | 2.382 μM·L^-1^ |
| **k^-^_TRPN_** | Dissociation rate of [Ca^2+^] to troponin | 0.045 s^-1^ |
| **k^+^_TRPN_** | Binding rate of [Ca^2+^] to troponin | 40 mM·L^-1^·s^-1^ |
| **[B]_TRPN_** | Total cytosolic troponin concentration | 0.07 mM·L^-1^ |

**Table S4. Initial conditions for state variables**

| **Variable** | **Definition** | **Initial value** |
| --- | --- | --- |
| **m** | I_Na_ activation gating variable | 0.0054828 |
| **h** | I_Na_ fast inactivation gating variable | 0.6095126 |
| **j** | I_Na_ slow inactivation gating variable | 0.60876276 |
| **r** | I_t_ activation gating variable | 0.002542 |
| **s** | I_t_ fast inactivation gating variable | 0.8823 |
| **s_slow_** | I_t_ slow inactivation gating variable | 0.42756 |
| **r_ss_** | I_ss_ activation gating variable | 0.002907171 |
| **s_ss_** | I_ss_ inactivation gating variable | 0.3142767 |
| **y** | inactivation gating variable | 3.578708e^-3^ |
| **z_1_** |  | 0.9886 |
| **z_2_** |  | 0.008873 |
| **z_3_** |  | 0.002366 |
| **V** | Membrane potential | -80 mV |
| **[Ca^2+^]_SR_** | SR Ca^2+^ concentration | 721.96 μM·L^-1^ |
| **[TRPN]** | Intracellular troponin concentration | 63.6364 μM·L^-1^ |
| **[Na^+^]_i_** | Intracellular Na^+^ concentration | 11.28 mM·L^-1^ |
| **[K^+^]_i_** | Intracellular K^+^ concentration | 138.7225 mM·L^-1^ |
| **[Ca^2+^]_i_** | Intracellular Ca^2+^ concentration | 0.11423 μM·L^-1^ |
| **T_ref_** | Reference tension | 120 mN·mm^-2^ |
| **K_on_** | Binding rate of [Ca^2+^] to troponin | 0.1 s^-1^ |
| **K_reoff_** | Unbinding rate in the absence of tension | 0.0001 s^-1^ |
| **γ** | measure of the affect of tension on the unbinding rate | 2 |
